# Generalization of Entropy Based Divergence Measures for Symbolic Sequence Analysis

**DOI:** 10.1371/journal.pone.0093532

**Published:** 2014-04-11

**Authors:** Miguel A. Ré, Rajeev K. Azad

**Affiliations:** 1 Departamento de Ciencias Básicas, CIII - Facultad Regional Córdoba, Universidad Tecnológica Nacional, Córdoba, Argentina; 2 Facultad de Matemática, Astronomía y Física, Universidad Nacional de Córdoba, Córdoba, Argentina; 3 Department of Biological Sciences, University of North Texas, Denton, Texas, United States of America; 4 Department of Mathematics, University of North Texas, Denton, Texas, United States of America; Technical University Darmstadt, Germany

## Abstract

Entropy based measures have been frequently used in symbolic sequence analysis. A symmetrized and smoothed form of Kullback-Leibler divergence or relative entropy, the Jensen-Shannon divergence (JSD), is of particular interest because of its sharing properties with families of other divergence measures and its interpretability in different domains including statistical physics, information theory and mathematical statistics. The uniqueness and versatility of this measure arise because of a number of attributes including generalization to any number of probability distributions and association of weights to the distributions. Furthermore, its entropic formulation allows its generalization in different statistical frameworks, such as, non-extensive Tsallis statistics and higher order Markovian statistics. We revisit these generalizations and propose a new generalization of JSD in the integrated Tsallis and Markovian statistical framework. We show that this generalization can be interpreted in terms of mutual information. We also investigate the performance of different JSD generalizations in deconstructing chimeric DNA sequences assembled from bacterial genomes including that of *E. coli*, *S. enterica typhi*, *Y. pestis* and *H. influenzae*. Our results show that the JSD generalizations bring in more pronounced improvements when the sequences being compared are from phylogenetically proximal organisms, which are often difficult to distinguish because of their compositional similarity. While small but noticeable improvements were observed with the Tsallis statistical JSD generalization, relatively large improvements were observed with the Markovian generalization. In contrast, the proposed Tsallis-Markovian generalization yielded more pronounced improvements relative to the Tsallis and Markovian generalizations, specifically when the sequences being compared arose from phylogenetically proximal organisms.

## Introduction

The statistical analysis of symbolic sequences is of great interest in diverse fields, such as, linguistics, image processing or biological sequence analysis. Information-theoretic measures based on Boltzmann-Gibbs-Shannon Entropy (BGSE) have been frequently used for interpreting discrete, symbolic data [1]. Using information-theoretic functionals makes it unnecessary to map the symbolic sequence to a numeric sequence. Given a random variable *X* with *k* possible values *e_i_*, *i*  = 1, 2, …, *k*, BGSE of the probability distribution 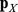 is defined as,
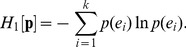
(1)


BGSE has an additivity property: Let *X* and *Y* be two statistically independent variables and 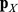 and 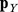
be their corresponding probability distributions so that their joint probability distribution is the product of their marginal distributions: 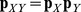. Then,
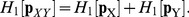
(2)


The central role played by BGSE in information theory has encouraged the proposals of generalization of this function. Outstanding in the realm of statistical physics has been the Tsallis generalization of BGSE [2], [3], which was obtained by substituting natural logarithm by its deformed expression [4],
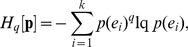
(3)with the deformed definition,
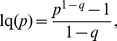
.where *q* is a real number and in the limit *q*→1, lq→ln and BGSE is recovered. Index *q* gives a measure of the non­extensivity of the generalization as expressed by the pseudo-additivity rule [2], [3]:
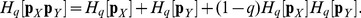
(4)In the limit q→1, the BGSE additivity as in eqn. 2 is recovered.

Measures based on BGSE have been proposed for measuring the difference between probability distributions. This includes the Kullback-Leibler divergence and its symmetrized forms [5]. Lin introduced the Jensen­Shannon divergence (JSD) as a generalization of a symmetrized version of Kulback­Leibler divergence, assigning weights to the probability distributions involved according to their relative importance [5]. Subsequently, different generalizations of JSD were proposed, either within the framework of Tsallis statistics [6] or within Markovian statistical framework [7]. While the former exploits the non-extensivity implicit in the Tsallis generalization of BGSE, the latter is based on conditional entropy that facilitates exploiting higher order correlations within symbolic sequences. Since the latter was obtained within the framework of Markov chain models, this generalization was named Markovian Jensen-Shannon divergence (MJSD) and was shown to significantly outperform standard JSD in its application to deciphering genomic heterogeneities [7], [8].

Because of the importance and usefulness of JSD in different disciplines, significant advances have been made in the generalization and interpretation of this measure. Yet a comprehensive treatise on generalization as well as comparative assessment of the generalized measures has remained elusive. Here, we have attempted to bridge the gaps by providing the missing details. Furthermore, we present here a non-extensive generalization of MJSD within the Tsallis statistical framework. The flexibility afforded by the integrated Tsallis-Markovian generalization has spawned new opportunities for (re-)visiting and exploring the symbolic sequence data prevalent in different domains. In the following section, we summarize the standard JSD, its properties and its interpretation in different contexts. This was leveraged to demonstrate in the next sections that certain interpretations are readily amenable to different generalizations of JSD including the proposed Tsallis-Markovian generalization. In section 3, we describe non-extensive JSD generalization, followed by conditional dependence based or Markovian generalization in section 4. In section 5, we propose a non-extensive generalization of the Markovian generalization of JSD. Finally, in section 6, we present a comparative assessment of the generalized measures in deconstructing chimeric DNA sequence constructs. Note also that in the following sections, for the sake of simplicity, we obtain the generalizations of JSD for two probability distributions or symbolic sequences. The generalization to any number of distributions or sequences is straightforward (as with the standard JSD, Eqn. 9 in section 2).

## Theory and Methods

### 1. The Jensen­Shannon Divergence Measure

Consider a discrete random variable *X* (with *k* possible values) and two probability distributions for *X*, *p_1_* and *p_2_*. The Kullback-Leibler information gain or Kullback-Leibler divergence (KLD) is defined as [1],
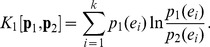
(5)KLD is not symmetric and requires absolute continuity (*p_1_*(*x_j_*)  = 0 when *p_2_*(*x_j_*)  = 0). To overcome these shortcomings, Lin [5] introduced a symmetrized generalization of KLD, namely, the L-divergence, defined as,
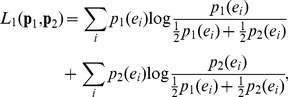
(6)which can be expressed in an entropic form, i.e.
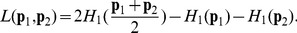
(7)The generalization of the L divergence is straightforward, defined as Jensen-Shannon divergence,
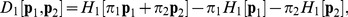
(8)where H1[.] is BGSE (Eqn. 1). The weights 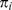 associated with the probability distributions pi allow assigning differential importance to each probability distribution. JSD does not require absolute continuity of probability distributions with respect to each other. Furthermore, JSD can be readily extended to include more than two probability distributions,



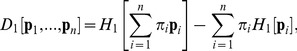
(9)given *n* probability distributions.

Being the natural logarithm of a concave function, JSD is non-negative, 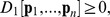
as can be verified from Jensen’s inequality. In addition to non-negativity and symmetricity, JSD also has a lower and upper bound, 0≤JSD≤1, and has been shown to be the square of a metric [6], [7], [9], [10]. Because of these interesting properties, this measure has been successfully applied to solving a variety of problems arising from different fields including molecular biology (e.g. DNA sequence analysis) [9], [11]–[17], condensed matter physics [18], atomic and molecular physics [19], and engineering (e.g. edge detection in digital imaging) [20].

Grosse *et al.* gave three intuitive interpretations of JSD in the framework of statistical physics, information theory and mathematical statistics [9]. Since we intend to show in the later sections that some of these interpretations could be readily extended to the generalized JSD measures, we briefly describe below the three interpretations of JSD.

#### Interpretation A (IA): Framework of statistical physics

In the framework of statistical physics, JSD can be interpreted as the intensive entropy of mixing. Considering two vessels with a mixture of ideal gases, the mixing entropy is obtained as,
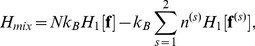
(10)where *k_B_* is Boltzmann constant, *s* is the number of vessels, *n* ^(*s*)^ denotes the number of gas particles in vessel *s*, 
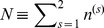
denotes the total number of ideal gas particles, **f** ^(*s*)^ denotes vector of molar fractions of the gases in vessel *s*, and 
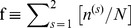
 **f** ^(*s*)^ denotes the vector of molar fractions of all gases in the mixture. Under this interpretation,
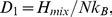
(11)identifying 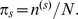 Given s subsequences, D1 could thus be interpreted as the overall difference between the entropy of the total sequence and the weighted average of the entropies of subsequences (each subsequence represented by a probability distribution, see Eqn. 9).

#### Interpretation B (IB): Framework of information theory

In the framework of information theory, *D_1_* can be interpreted as the mutual information. Consider two subsequences *S*_1_, *S*_2_ of length *n*_1_ and *n*_2_ symbols respectively, derived from an alphabet *A*  =  {*e*_1_, …, *e_k_*}of *k* symbols. The mutual information of symbols and the subsequences they belong to (denoted *E* and *S* respectively, representing all symbols and all subsequences) is given as,
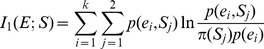
(12)

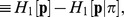
.which is the reduction in the uncertainty of *E* due to the knowledge of *S*. Here, *p* (*e_i_*, *S_j_*) is the joint probability of variables *e_i_* and *S_j_*. The marginal probabilities 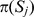
and 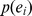
are defined as,
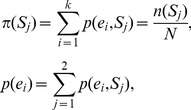
(13)and the conditional entropy 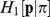
is defined as,
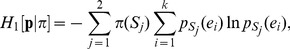
(14)where the conditional probability
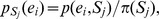
which is the probability of finding symbol ei in the given subsequence Sj. Mutual information can be rewritten as,
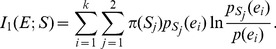
(15)Recognizing 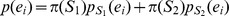
in this last expression, we re-obtain (8)

#### Interpretation C (IC): Framework of mathematical statistics

In the framework of mathematical statistics, *D_1_* can be interpreted as the log­likelihood ratio. Consider the sequence *S* composed of *N* symbols as in IB but we now ask for the probability distribution **p** that maximizes the likelihood of *S*. The maximum likelihood principle suggests.



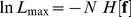
(16)with 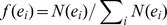, *i.e.* the relative frequency of symbol *e_i_* in the sequence *S*. The probability distribution that maximizes the likelihood is **p** = **f**. A similar calculation can be carried out for the likelihood of subsequences *S_j_* composing the sequence *S*. Under this interpretation, we have,
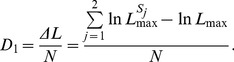
(17)Here, ΔL is the log­likelihood ratio which gives a measure of the increase in the log­likelihood when sequence S is modeled as a concatenation of two subsequences.

### 2. Non­extensive Generalization of JSD

Several forms of generalization in terms of non-extensive entropy (Eqn. 3), introduced by Tsallis in modeling physical systems with long range interactions [3], have been suggested. The different JSD generalizations found in the literature can be interpreted under the schema presented in the previous section as IA or IB. A key concept in these generalizations is that of mutual information measure.

Burbea and Rao [21] defined a generalized mutual information measure via entropy substitution, which may be interpreted as in IA. The generalized JSD can be obtained by merely substituting *H_1_* by *H_q_* in Eqn. 8:
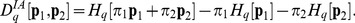
(18)An alternative generalization was obtained by Lamberti and Majtey [6] via the non-extensive generalization of KL divergence proposed by Tsallis [22]:
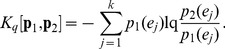
(19)The symmetrized L-divergence, in the framework of Tsallis statistics, was obtained as,
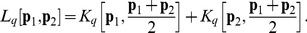
(20)The Lq-divergence was shown to generalize to JSq-divergence, replacing equal weights for the two distributions with any arbitrary weights π1 and π2 associated with p1 and p2. However, this generalization does not assume full entropic form as 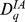 [6]:



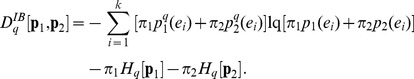
(21)Jensen’s inequality allows to show that 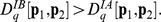 We have put the supraindex *IB* in the former as this generalization has an interpretation in mutual information. 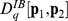
can be rewritten as,
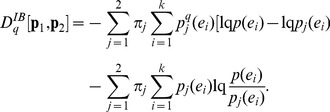
(22)Expression (22) can be interpreted as mutual information in Tsallis non­extensive statistics, being a generalization of Eqn. (15):
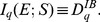
(23)As noted in [22], Iq (E; S) gives a measure of the independence of two random variables: Iq (E; S) = 0 for independent variables. In this case of statistically independent variables, the probability distribution of symbols ei is the same for both sequence segments. Here, S is interpreted as a random variable with probability distribution given by the weights πj.

### 3. Markov Model Generalization of JSD

The standard JSD measure assumes each symbol in a sequence to occur independent of the others. In order to account for short range interdependence between symbols, JSD can be generalized by means of conditional entropy. This generalization can be obtained in the framework of Markov chain model of order *m*, where the occurrence of a symbol is dependent on the *m* preceding symbols in the sequence. The JSD corresponding to Markov sources can be obtained following the steps in the derivation of JSD (Eqn. 6–8) for the independent and identically-distributed *(i.i.d*.) sources. For example, for a Markov source of order *m*, where the occurrence of symbol *e_i_* depends on its just preceding context *w* of length *m*,
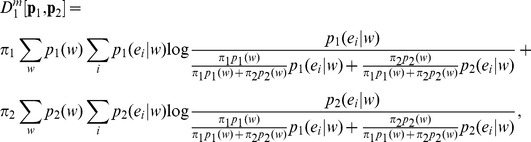
(24)which leads to, after rearranging,
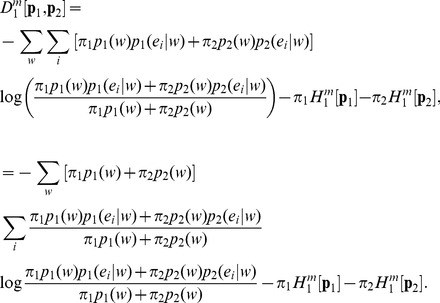
(25)Therefore,
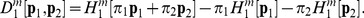
(26)Here 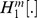
corresponds to entropy function for Markov sources of order m,



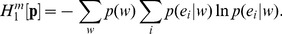
(27)In contrast to Lamberti and Majtey’s generalization within the Tsallis non-extensive statistical framework [6] (Eqn. 21), this generalization takes the full entropic form. Thakur et al. introduced “Markov models for genomic segmentation” (MMS) [7], where they replaced the BGSE with Markovian entropy (Eqn. 27) in the expression of JSD (Eqn. 8), which is amenable to interpretation IA. They also derived this generalization, which we call Markovian JSD (MJSD) introduced earlier in [8], using the likelihood function (interpretation IC).

This generalization could also be interpreted in terms of conditional mutual information, consistent with interpretation IB (Eqn. 15),
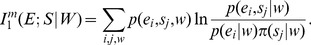
(28)


Making use of the conditional entropy definition and after some algebraic manipulation, one can identify 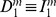
according to interpretation IB.

### 4. Non-extensive Markovian JSD Generalization

We obtain the generalization of MJSD within the framework of Tsallis non-extensive statistics. This integrates two different generalizations of JSD, the Markovian and the Tsallis generalization, thus yielding a generalization of which many of the previously described JSD generalizations are special cases.

The non-extensive conditional or Markovian Kullback-Leibler divergence between two distributions **p_1_** and **p_2_** is defined as:
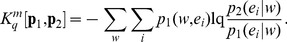
(29)Using the above, the symmetrized *L*-divergence in Tsallis-Markovian framework can thus be obtained as,
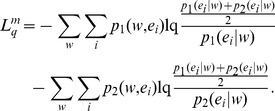
(30)Thus, we get,
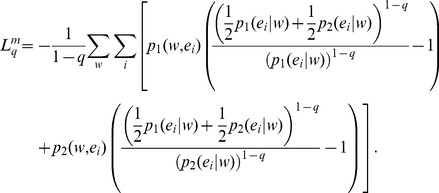
(31)Rearranging,
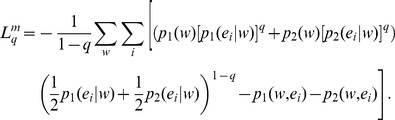
(32)Therefore,



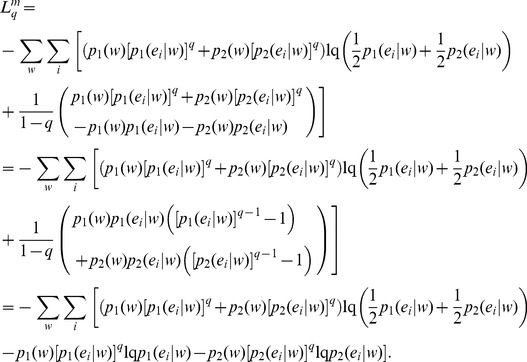
(33)The Tsallis-Markovian generalization for equal weights for the two distributions **p_1_** and **p_2_** (*π_1_* = 0.5, *π_2_* = 0.5) could thus be expressed as,
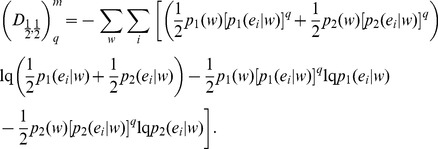
(34)The generalization to any weights 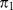 and 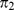 (from 
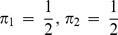
) associated to the joint distributions 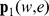 and 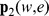 respectively is straightforward:



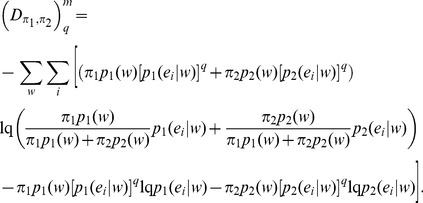
(35)Note that the above generalization does not take an entropic form or admit replacement of BGSE with non-extensive conditional entropy in Eqn. 8 or 11 (interpretation IA), however, it can be interpreted as mutual information (interpretation IB) as demonstrated below.

Beginning with the conditional mutual information,
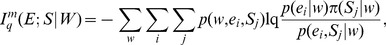
(36)we identify, as in *q* = 1 cases (Eqns. 15 and 28), that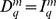.

If conditional probabilities 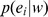
and 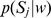
are independent, then
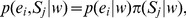
(37)and in this situation, 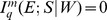, so that the conditional mutual information is a measure of the independence of the conditional probabilities.

Eqn. (36) can be rewritten as, by means of lq definition,
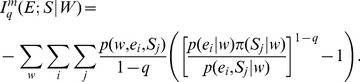
(38)By means of Bayes’ theorem,
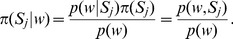
(39)We may rewrite,
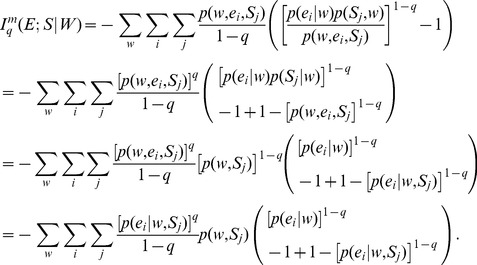
(40)And, therefore, the generalization can be obtained as,
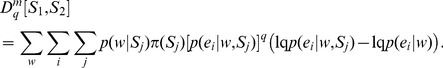
(41)Notice that for model order 0, Eqn. 41 reduces to Lamberti and Majtey’s non-extensive generalization [6] (Eqn. 21), while in the limit q→1, we recover Thakur et al.’s Markovian generalization [7]. Note that 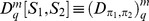 (Eqn. 35) and therefore, the Tsallis-Markovian generalization of JSD has its interpretation in mutual information.

## Experiments and Assessment

To assess the discriminative abilities of JSD and its generalized forms, we compiled a test set of chimeric sequence constructs by concatenating DNA sequences from phylogenetically distinct organisms. Let *S* be a sequence composed of symbols *e_i_* from an alphabet of *k* symbols (*i* = 1,…,*k*). Let us further assume that sequence *S* is the concatenation of two subsequences *S_1_* and *S_2_*. Let 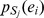 denote the probability of symbol *e_i_* in subsequence *S_j_*, and *p*(*S_j_*), or simply *π_j_*, the weight associated with the distribution **p_j_** (j = 1,2). Since the actual probability
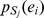
is often not known, the relative frequency of symbol *e_i_* in subsequence *S_j_*, 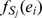, is used as the estimate of 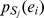. Thus, *D*_1_ [**p**_1_, **p**_2_] or its generalizations for given subsequences *S_1_* and *S_2_* is, in effect, a measure of the difference between the estimates of **p**_1_ and **p**_2_. We use weights *π_j_* proportional to the length of *S_j_*, which was earlier found to be most appropriate for symbolic sequence analysis [9].

Chimeric sequence constructs were obtained by concatenating two equal size sequence segments selected randomly from the genomes of two different organisms. We chose four phylogenetically distinct organisms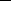 *Escherichia coli*, *Salmonella enterica*, *Yersinia pestis* and *Haemophilus influenzae*, the first three belongs to the family *Enterobacteriaceae* and the fourth is an outgroup belonging to the family *Pasteurellaceae*. We obtained the sequence constructs of 20 Kbp by concatenating 10 Kbp genomic segment from *E. coli* with 10 Kbp segment from one of the other three organisms. The phylogenetic proximity of these organisms from *E. coli* is in the following order: *S. enterica* > *Y. pestis* > *H. influenzae*. We subjected the non-extensive MJSD to detecting the join point of the two disparate sequence segments. A cursor was moved along the chimeric sequence construct and the non-extensive MJSD was computed for sequence segments left and right to the cursor. The position where non-extensive MJSD was maximized was noted. The error in localizing the join point was obtained as the absolute difference between the position where the non-extensive MJSD was maximized and the position of the join point in a sequence construct (for sequence constructs of 20 Kbp, the maximum and minimum possible error would thus be 10,000 bp and 0 bp respectively).

**Figure 1:**
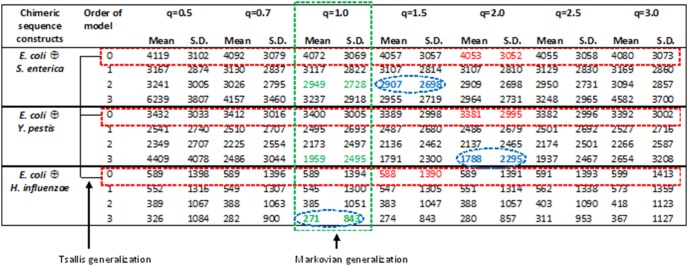
Error (in base pairs) in detecting the join point in the chimeric sequence constructs for *E. coli* 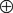 *S. enterica*, *E. coli* 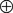 *Y. pestis*, and *E. coli* 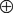 *H. influenzae*(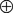
denotes concatenation). The proposed Tsallis-Markovian generalization of the Jensen-Shannon divergence measure was used to obtain the mean and standard deviation of the error from 10,000 replicates for each type of chimeric sequence constructs. The error in localizing the join point was obtained as the absolute difference between the position where the divergence was maximized and the position of the join point (at 10 Kbp) in a chimeric sequence construct of size 20 Kbp. Error statistics for the two special cases of the proposed generalized measure is shown within rectangular boxes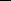 the Markovian generalization (*q* = 1) in dashed green border box and Tsallis non-extensive generalization (model order = 0) in dashed red border boxes. The minimum values of mean and standard deviation of the error for each chimeric construct type are shown encircled and bold faced.

**Figure 2:**
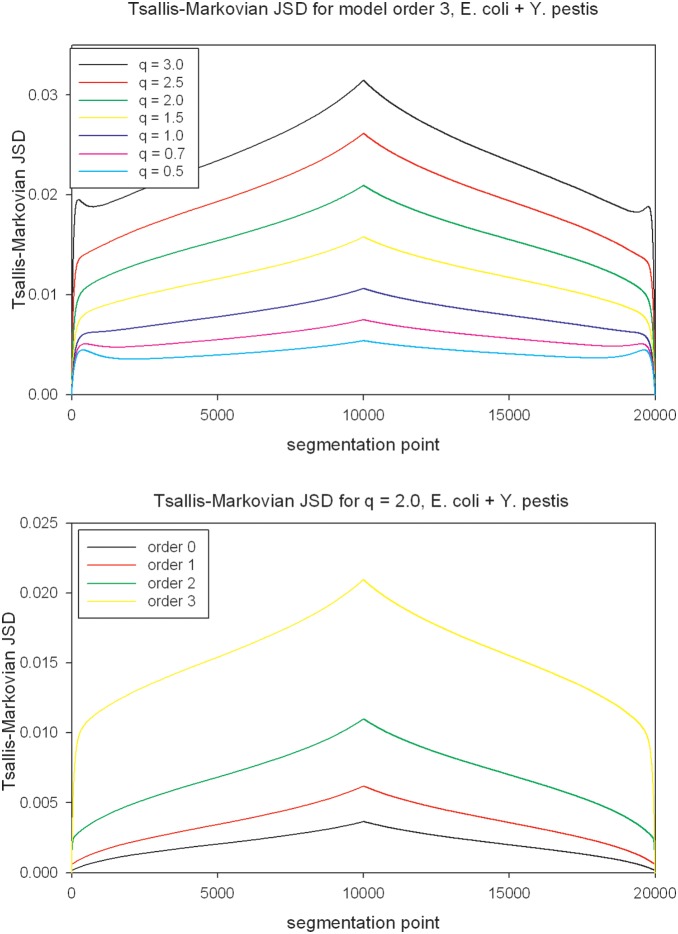
Mean values of non-extensive MJSD at each position of the chimeric sequence constructs *E. coli* 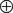 *Y. pestis*, for the parameter setting at which the non-extensive MJSD achieved most pronounced error reduction (*q* = 2, order 3). The chimeric constructs of size 20 Kbp are comprised of two equal sized sequences, with each component sequence of length 10 Kbp obtained from the genome of each organism.

**Figure 3:**
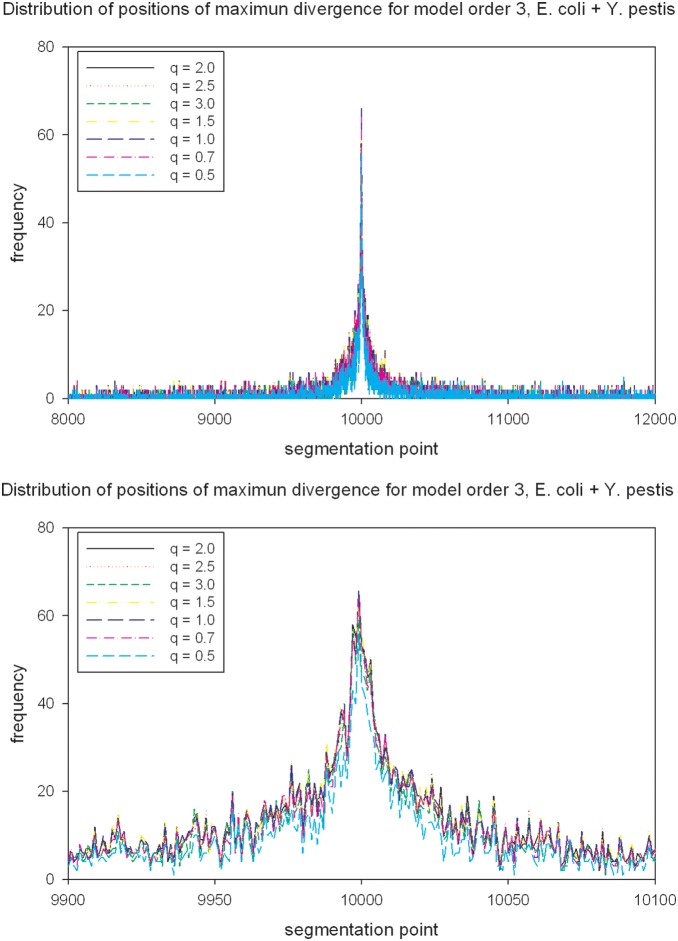
Frequency distribution of position with maximum value of non-extensive MJSD for the chimeric sequence constructs *E. coli* 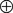 *Y. pestis*, for the parameter setting at which the non-extensive MJSD achieved most pronounced error reduction (*q* = 2, order 3). The chimeric constructs of size 20 Kbp are comprised of two equal sized sequences, with each component sequence of length 10 Kbp obtained from the genome of each organism.

For experiments with 10,000 replicates for each, *E. coli* 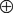 *S. enterica, E. coli* 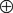 *Y. pestis,* and *E. coli* 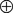 *H. influenzae* (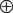
denotes concatenation), the mean errors in detecting the join point for standard JSD (*q* = 1, order 0) were 4072, 3400 and 589 bp respectively, consistent with the order of divergence of *E. coli* from the other three organisms, with *H. influenzae* being the outgroup (Figure 1). For the non-extensive generalization (*q* varies, order 0; error statistics shown within three rectangular boxes with dashed red borders in Figure 1), the minimum mean errors (in the same order of divergence from *E. coli*) were observed to be 4053, 3381 and 588 bp for *q* in the range 1.5−2.0. Since *H. influenzae* is phylogenetically distant from *E. coli*, the generalization induces very minor improvement while for the others, all belonging to the same family, the generalization induces more improvement apparently due to more rooms for improvement in these cases. In contrast, for the Markovian generalization (*q* = 1, order varies; error statistics shown within rectangular box with dashed green borders in Figure 1), the improvements were substantially more pronounced with corresponding minimum mean errors being 2949, 1959 and 271 bp at order 2, 3 and 3 respectively. This large improvement is apparently due to the Markovian generalization accounting for short-range correlations in the nucleotide ordering in genomic sequences, which is not considered in the non-extensive generalization. As expected from the above results, the non-extensive Markovian generalization induces further improvement over the Markovian generalization, generating the respective minimum mean errors of 2907, 1788 and 271 bp at different combinations of *q* and model order (shown encircled and bold faced in Figure 1). Clearly, the non-extensive generalization reaches saturation in improvement at large phylogenetic distances between the organisms under comparison while it induces significant improvements for phylogenetically proximal organisms. Indeed, the reduction of more than 40 bp in error for *E. coli* 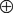 *S. enterica* and 170 bp for *E. coli* 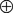 *Y. pestis* is remarkable considering that these organisms are phylogenetically very close and therefore difficult to differentiate in their genomic composition [13]. The higher values of standard deviation from the mean are likely because of the non-homogeneity of the bacterial genomes. A significant portion (∼1–20%) of bacterial DNAs is mobile and therefore distinct from the ancestral DNAs acquired though the reproductive processes [23]. The mean values of non-extensive MJSD at each position of the chimeric sequence constructs *E. coli* 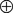 *Y. pestis* and the frequency distribution of position with maximum value of non-extensive MJSD for these sequence constructs are shown in Figure 2 and Figure 3 respectively, for the parameter setting at which the non-extensive MJSD achieved most pronounced error reduction (*q* = 2, order 3). Notably, the value of MJSD increases monotonically with increase in *q* or model order or both (Figure 2). A sharp spike in the distribution around position 10 Kbp demonstrates the efficiency of the divergence measure in localizing the join point of *E. coli* and *Y. pestis* sequences (Figure 3), with the best performance at *q* = 2 and order 3 setting (Figure 1). We show in Figures S1–S15 these data for all three kinds of sequence construct and at all parameter settings.

In Figure S16, we show the error statistics for cases when the chimeric sequence constructs of 20 Kbp had 5 Kbp from a non-*E. coli* organism (*S. enterica, Y. pestis* or *H. influenzae*) and the remaining 15 Kbp from *E. coli*. The variable length taxonomically distinct sequences within chimeric constructs present significantly more challenge for the statistical methods than the chimeric constructs with similar size sequences. As expected, the mean errors in detecting the join point increased in all cases. The Markovian generalization still results in much better performance than the non-extensive generalization, while the non-extensive Markovian generalization led to a more pronounced improvement for *E. coli* 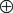 *Y. pestis* (a reduction of 295 bp in mean error compared with the Markovian generalization). Non-extensive generalization of MJSD didn’t induce further improvement for *E. coli* 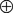 *S. enterica*, likely because of the weakened discriminatory signal as a consequence of reduction in the size of *S. enterica* fragments. Figures S17–S31 provide plots for divergence values at each sequence position as well as frequency distributions of position with maximum divergence for all three kinds of sequence construct and at all parameter settings. The discrimination of DNA sequences from phylogenetically close relatives such as *E. coli* and *S. enterica* is difficult, yet this study shows that there are still rooms for improvement with the development of more flexible, sensitive methods. Overall, the non-extensive Markovian generalization results in improved efficiency in discriminating sequences from phylogenetically proximal organisms.

## Conclusions

The proposed generalization of JSD in the integrated framework of Tsallis and Markovian statistics provides a powerful tool for symbolic sequence analysis. In application to deconstructing the chimeric bacterial sequences, the Tsallis-Markovian generalization achieved remarkable improvement over both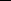 the Tsallis as well as the Markovian generalization. The superior performance of Tsallis-Markovian JSD was most pronounced when the sequences under comparison arose from phylogenetically proximal organisms. *E. coli*, *S. enterica* and *Y. pestis*, all belong to the same *Enterobacteriaceae* family; previous studies have shown the limitations of JSD in distinguishing sequences from organisms belonging to the same family [13]. Therefore, the improvement achieved by the proposed generalized measure is an important step forward in interpreting the biological data which often have heterogeneities at varying levels. While for the first time, to the best of our knowledge, the theoretically distinct generalizations of JSD accomplished by different research groups have been brought to one place for comparison and assessment, this study has also bridged the gaps in the field by obtaining generalizations consistent with the original proposal for JSD derivation and by providing the interpretations in the framework of statistical physics, information theory and mathematical statistics, where possible. The proposed divergence measure, generalized in the integrated framework of Tsallis and Markovian statistics, provides a new exploratory tool, augmented in both power and flexibility, to mine the symbolic sequence data.

## Supporting Information

Figure S1Mean values of non-extensive MJSD at each position of the chimeric sequence constructs *E. coli* 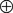 *S. enterica*, for model order *m* = 0–3. For each model order, plots are shown for different values of Tsallis statistics’ parameter *q*, in the range 0.5–3. The chimeric constructs of size 20 Kbp are comprised of two equal sized sequences, with each component sequence of length 10 Kbp obtained from the genome of each organism.(TIF)

Figure S2Mean values of non-extensive MJSD at each position of the chimeric sequence constructs *E. coli* 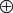 *S. enterica*, for Tsallis statistics’ parameter *q* = 0.5, 0.7, 1.0, 1.5. For each *q*, plots are shown for different model orders, in the range 0–3. The chimeric constructs of size 20 Kbp are comprised of two equal sized sequences, with each component sequence of length 10 Kbp obtained from the genome of each organism.(TIF)

Figure S3 As in Figure S2, but for Tsallis statistics’ parameter *q* = 2.0, 2.5, 3.0.
(TIF)

Figure S4Mean values of non-extensive MJSD at each position of the chimeric sequence constructs *E. coli* 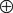 *Y. pestis*, for model order *m* = 0–3. For each model order, plots are shown for different values of Tsallis statistics’ parameter *q*, in the range 0.5–3. The chimeric constructs of size 20 Kbp are comprised of two equal sized sequences, with each component sequence of length 10 Kbp obtained from the genome of each organism.(TIF)

Figure S5Mean values of non-extensive MJSD at each position of the chimeric sequence constructs *E. coli* 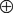 *Y. pestis*, for Tsallis statistics’ parameter *q* = 0.5, 0.7, 1.0, 1.5. For each *q*, plots are shown for different model orders, in the range 0–3. The chimeric constructs of size 20 Kbp are comprised of two equal sized sequences, with each component sequence of length 10 Kbp obtained from the genome of each organism.(TIF)

Figure S6 As in Figure S5, but for Tsallis statistics’ parameter *q* = 2.0, 2.5, 3.0.
(TIF)

Figure S7Mean values of non-extensive MJSD at each position of the chimeric sequence constructs *E. coli* 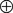 *H. influenzae*, for model order *m* = 0–3. For each model order, plots are shown for different values of Tsallis statistics’ parameter *q*, in the range 0.5–3. The chimeric constructs of size 20 Kbp are comprised of two equal sized sequences, with each component sequence of length 10 Kbp obtained from the genome of each organism.(TIF)

Figure S8Mean values of non-extensive MJSD at each position of the chimeric sequence constructs *E. coli* 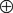 *H. influenzae*, for Tsallis statistics’ parameter *q* = 0.5, 0.7, 1.0, 1.5. For each *q*, plots are shown for different model orders, in the range 0–3. The chimeric constructs of size 20 Kbp are comprised of two equal sized sequences, with each component sequence of length 10 Kbp obtained from the genome of each organism.(TIF)

Figure S9 As in Figure S8, but for Tsallis statistics’ parameter *q* = 2.0, 2.5, 3.0.
(TIF)

Figure S10Frequency distribution of position with maximum value of non-extensive MJSD for the chimeric sequence constructs *E. coli* 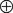 *S. enterica*, for model order *m* = 0 (A, B) and 1 (C, D). For each model order, distributions are shown for different values of Tsallis statistics’ parameter *q*, in the range 0.5–3. The chimeric constructs of size 20 Kbp are comprised of two equal sized sequences, with each component sequence of length 10 Kbp obtained from the genome of each organism.(TIF)

Figure S11 As in Figure S10, but for model order *m* = 2 (E, F) and 3 (G, H).
(TIF)

Figure S12Frequency distribution of position with maximum value of non-extensive MJSD for the chimeric sequence constructs *E. coli* 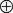 *Y. pestis*, for model order *m* = 0 (A, B) and 1 (C, D). For each model order, distributions are shown for different values of Tsallis statistics’ parameter *q*, in the range 0.5–3. The chimeric constructs of size 20 Kbp are comprised of two equal sized sequences, with each component sequence of length 10 Kbp obtained from the genome of each organism.(TIF)

Figure S13 As in Figure S12, but for model order *m* = 2 (E, F) and 3 (G, H).
(TIF)

Figure S14Frequency distribution of position with maximum value of non-extensive MJSD for the chimeric sequence constructs *E. coli* 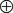 *H. influenzae*, for model order *m* = 0 (A, B) and 1 (C, D). For each model order, distributions are shown for different values of Tsallis statistics’ parameter *q*, in the range 0.5–3. The chimeric constructs of size 20 Kbp are comprised of two equal sized sequences, with each component sequence of length 10 Kbp obtained from the genome of each organism.(TIF)

Figure S15 As in Figure S14, but for model order *m* = 2 (E, F) and 3 (G, H).
(TIF)

Figure S16Error (in base pairs) in detecting the join point in the chimeric sequence constructs for *E. coli* 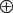 *S. enterica, E. coli* 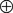 *Y. pestis*, and *E. coli* 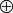 *H. influenzae* (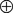
denotes concatenation). The proposed Tsallis-Markovian generalization of the Jensen-Shannon divergence measure was used to obtain the mean and standard deviation of the error from 5,000 replicates for each type of chimeric sequence constructs. The error in localizing the join point was obtained as the absolute difference between the position where the divergence was maximized and the position of the join point (at 5 Kbp) in a chimeric sequence construct of size 20 Kbp (5 Kbp sequence from non-*E. coli* organism concatenated with 15 Kbp from *E. coli*). Error statistics for the two special cases of the proposed generalized measure is shown within rectangular boxes– the Markovian generalization (*q* = 1) in dashed green border box and Tsallis non-extensive generalization (model order = 0) in dashed red border boxes. The minimum values of mean and standard deviation of the error for each chimeric construct type are shown encircled and bold faced.(TIFF)

Figure S17Mean values of non-extensive MJSD at each position of the chimeric sequence constructs *E. coli* 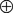 *S. enterica*, for model order *m* = 0–3. For each model order, plots are shown for different values of Tsallis statistics’ parameter *q*, in the range 0.5–3. The chimeric constructs of size 20 Kbp are comprised of two sequences, one component sequence of length 5 Kbp obtained from the genome of *S. enterica* and the other of length 15 Kbp from the genome of *E. coli*.(TIF)

Figure S18Mean values of non-extensive MJSD at each position of the chimeric sequence constructs *E. coli* 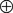 *S. enterica*, for Tsallis statistics’ parameter *q* = 0.5, 0.7, 1.0, 1.5. For each *q*, plots are shown for different model orders, in the range 0–3. The chimeric constructs of size 20 Kbp are comprised of two sequences, one component sequence of length 5 Kbp obtained from the genome of *S. enterica* and the other of length 15 Kbp from the genome of *E. coli*.(TIF)

Figure S19 As in Figure S18, but for Tsallis statistics’ parameter *q* = 2.0, 2.5, 3.0.
(TIF)

Figure S20Mean values of non-extensive MJSD at each position of the chimeric sequence constructs *E. coli* 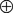 *Y. pestis*, for model order *m* = 0–3. For each model order, plots are shown for different values of Tsallis statistics’ parameter *q*, in the range 0.5–3. The chimeric constructs of size 20 Kbp are comprised of two sequences, one component sequence of length 5 Kbp obtained from the genome of *Y. pestis* and the other of length 15 Kbp from the genome of *E. coli*.(TIF)

Figure S21Mean values of non-extensive MJSD at each position of the chimeric sequence constructs *E. coli* 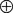 *Y. pestis*, for Tsallis statistics’ parameter *q* = 0.5, 0.7, 1.0, 1.5. For each *q*, plots are shown for different model orders, in the range 0–3. The chimeric constructs of size 20 Kbp are comprised of two sequences, one component sequence of length 5 Kbp obtained from the genome of *Y. pestis* and the other of length 15 Kbp from the genome of *E. coli*.(TIF)

Figure S22 As in Figure S21, but for Tsallis statistics’ parameter *q* = 2.0, 2.5, 3.0.
(TIF)

Figure S23Mean values of non-extensive MJSD at each position of the chimeric sequence constructs *E. coli* 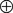 *H. influenzae*, for model order *m* = 0–3. For each model order, plots are shown for different values of Tsallis statistics’ parameter *q*, in the range 0.5–3. The chimeric constructs of size 20 Kbp are comprised of two sequences, one component sequence of length 5 Kbp obtained from the genome of *H. influenzae* and the other of length 15 Kbp from the genome of *E. coli*.(TIF)

Figure S24Mean values of non-extensive MJSD at each position of the chimeric sequence constructs *E. coli* 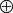 *H. influenzae*, for Tsallis statistics’ parameter *q* = 0.5, 0.7, 1.0, 1.5. For each *q*, plots are shown for different model orders, in the range 0–3. The chimeric constructs of size 20 Kbp are comprised of two sequences, one component sequence of length 5 Kbp obtained from the genome of *H. influenzae* and the other of length 15 Kbp from the genome of *E. coli*.(TIF)

Figure S25 As in Figure S24, but for Tsallis statistics’ parameter *q* = 2.0, 2.5, 3.0.
(TIF)

Figure S26Frequency distribution of position with maximum value of non-extensive MJSD for the chimeric sequence constructs *E. coli* 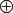 *S. enterica*, for model order *m* = 0 (A, B) and 1 (C, D). For each model order, distributions are shown for different values of Tsallis statistics’ parameter *q*, in the range 0.5–3. The chimeric constructs of size 20 Kbp are comprised of two sequences, one component sequence of length 5 Kbp obtained from the genome of *S. enterica* and the other of length 15 Kbp from the genome of *E. coli*.(TIF)

Figure S27 As in Figure S26, but for model order *m* = 2 (E, F) and 3 (G, H).
(TIF)

Figure S28Frequency distribution of position with maximum value of non-extensive MJSD for the chimeric sequence constructs *E. coli* 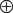 *Y. pestis*, for model order *m* = 0 (A, B) and 1 (C, D). For each model order, distributions are shown for different values of Tsallis statistics’ parameter *q*, in the range 0.5–3. The chimeric constructs of size 20 Kbp are comprised of two sequences, one component sequence of length 5 Kbp obtained from the genome of *Y. pestis* and the other of length 15 Kbp from the genome of *E. coli*.(TIF)

Figure S29 As in Figure S28, but for model order *m* = 2 (E, F) and 3 (G, H).
(TIF)

Figure S30Frequency distribution of position with maximum value of non-extensive MJSD for the chimeric sequence constructs *E. coli* 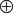 *H. influenzae*, for model order *m* = 0 (A, B) and 1 (C, D). For each model order, distributions are shown for different values of Tsallis statistics’ parameter *q*, in the range 0.5–3. The chimeric constructs of size 20 Kbp are comprised of two sequences, one component sequence of length 5 Kbp obtained from the genome of *H. influenzae* and the other of length 15 Kbp from the genome of *E. coli*.(TIF)

Figure S31 As in Figure S30, but for model order *m* = 2 (E, F) and 3 (G, H).
(TIF)
