# Supplementary figures and images for: Generalization of Entropy Based Divergence Measures for Symbolic Sequence Analysis
Source: PLoS One. 2014 Apr 11;9(4):e93532. doi: 10.1371/journal.pone.0093532 (PMC3984095; doi:10.1371/journal.pone.0093532)

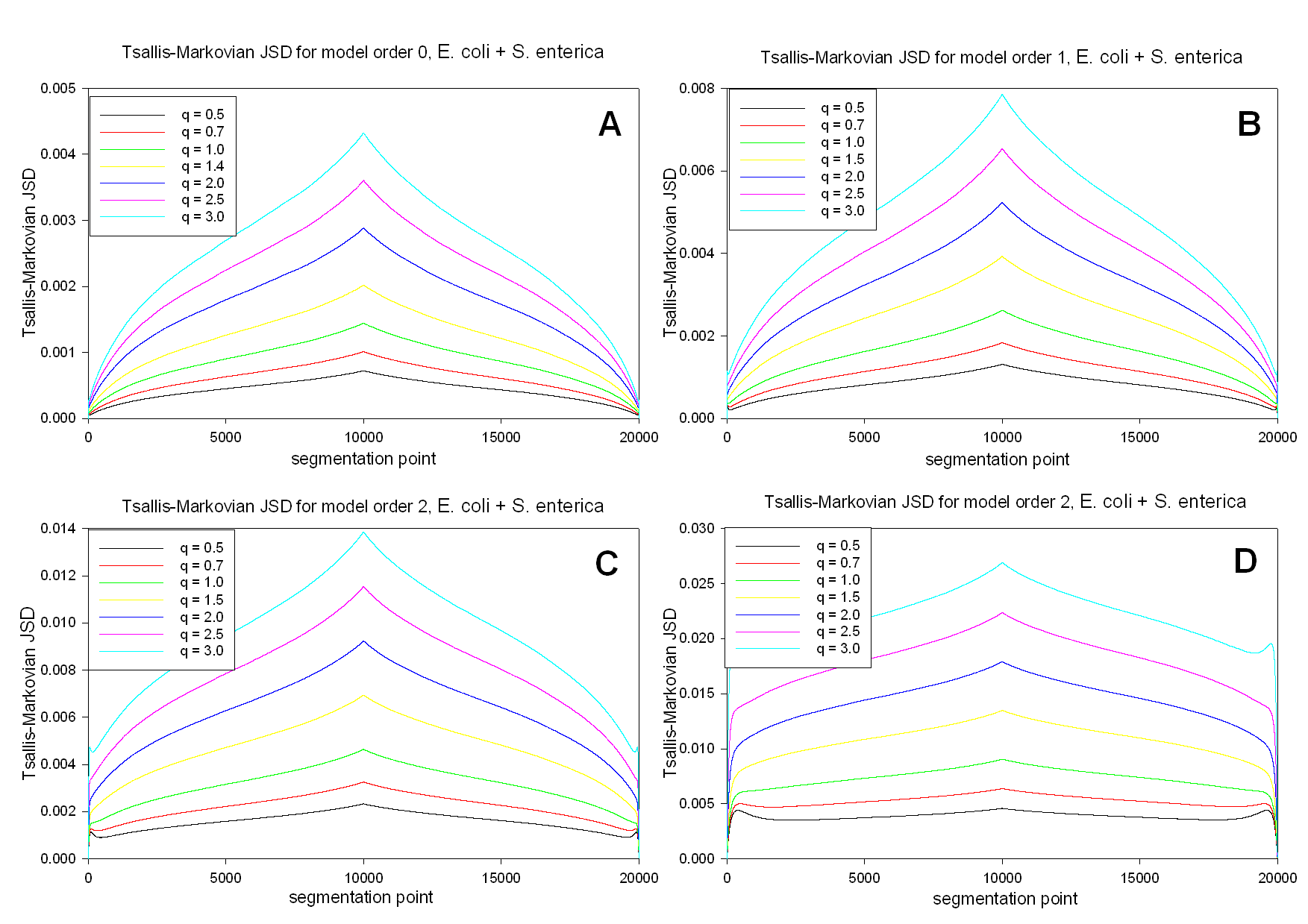

Supplement: Figure S1 — Mean values of non-extensive MJSD at each position of the chimeric sequence constructs E. coli S. enterica, for model order m = 0–3. For each model order, plots are shown for different values of Tsallis statistics’ parameter q, in the range 0.5–3. The chimeric constructs of size 20 Kbp are comprised of two equal sized sequences, with each component sequence of length 10 Kbp obtained from the genome of each organism. (TIF) [file pone.0093532.s001.tif]

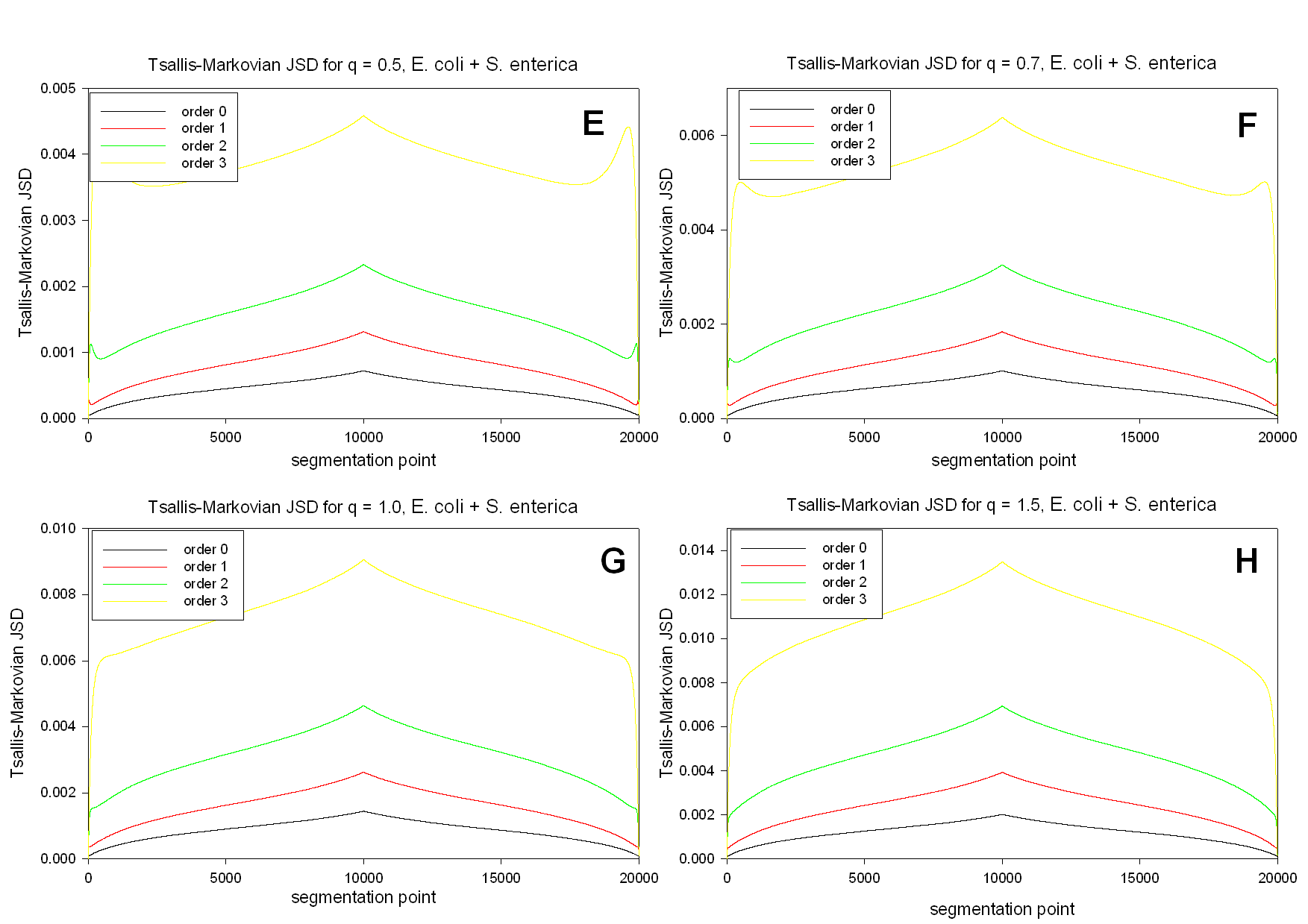

Supplement: Figure S2 — Mean values of non-extensive MJSD at each position of the chimeric sequence constructs E. coli S. enterica, for Tsallis statistics’ parameter q = 0.5, 0.7, 1.0, 1.5. For each q, plots are shown for different model orders, in the range 0–3. The chimeric constructs of size 20 Kbp are comprised of two equal sized sequences, with each component sequence of length 10 Kbp obtained from the genome of each organism. (TIF) [file pone.0093532.s002.tif]

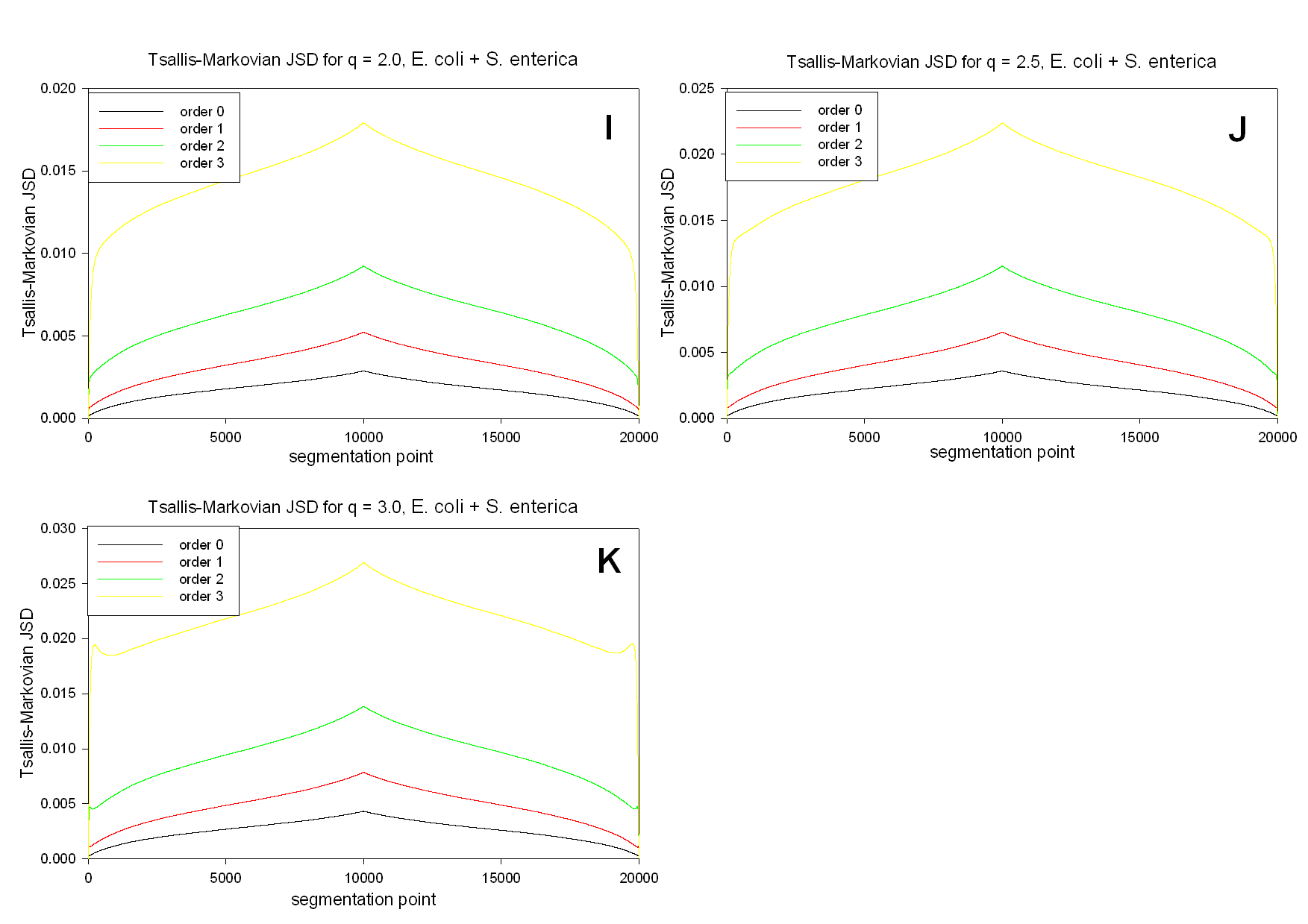

Supplement: Figure S3 — As in Figure S2, but for Tsallis statistics’ parameter q = 2.0, 2.5, 3.0. (TIF) [file pone.0093532.s003.tif]

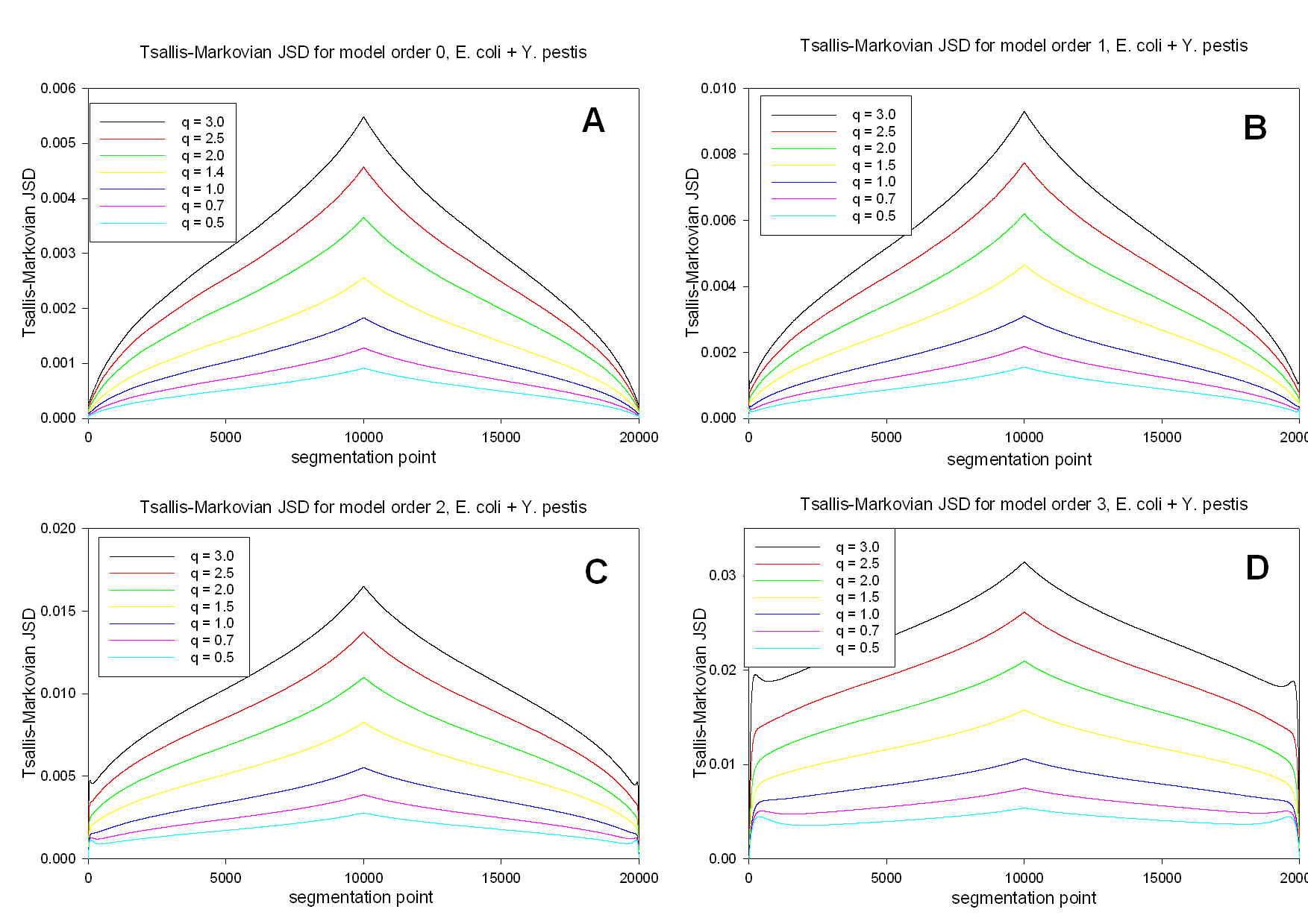

Supplement: Figure S4 — Mean values of non-extensive MJSD at each position of the chimeric sequence constructs E. coli Y. pestis, for model order m = 0–3. For each model order, plots are shown for different values of Tsallis statistics’ parameter q, in the range 0.5–3. The chimeric constructs of size 20 Kbp are comprised of two equal sized sequences, with each component sequence of length 10 Kbp obtained from the genome of each organism. (TIF) [file pone.0093532.s004.tif]

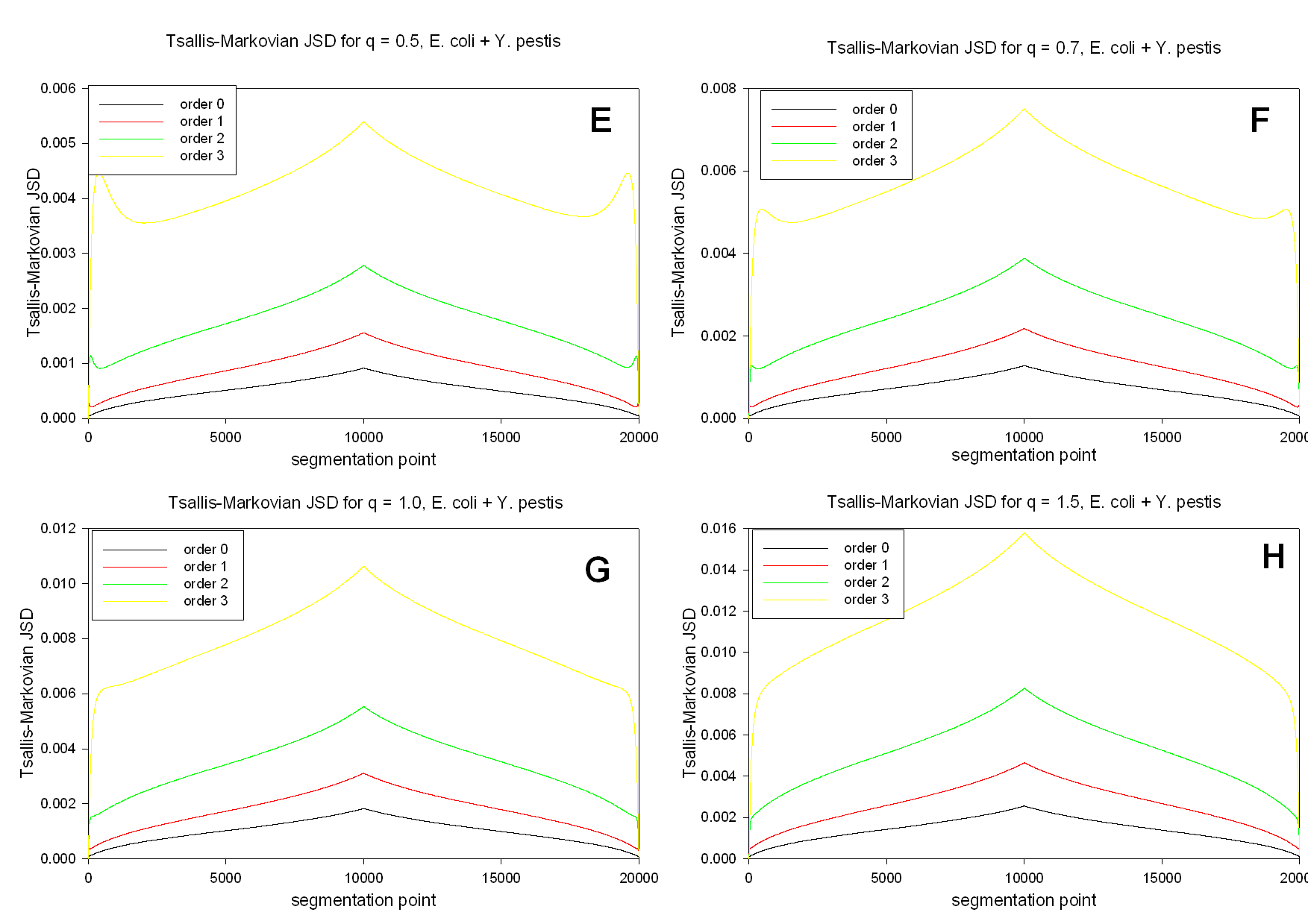

Supplement: Figure S5 — Mean values of non-extensive MJSD at each position of the chimeric sequence constructs E. coli Y. pestis, for Tsallis statistics’ parameter q = 0.5, 0.7, 1.0, 1.5. For each q, plots are shown for different model orders, in the range 0–3. The chimeric constructs of size 20 Kbp are comprised of two equal sized sequences, with each component sequence of length 10 Kbp obtained from the genome of each organism. (TIF) [file pone.0093532.s005.tif]

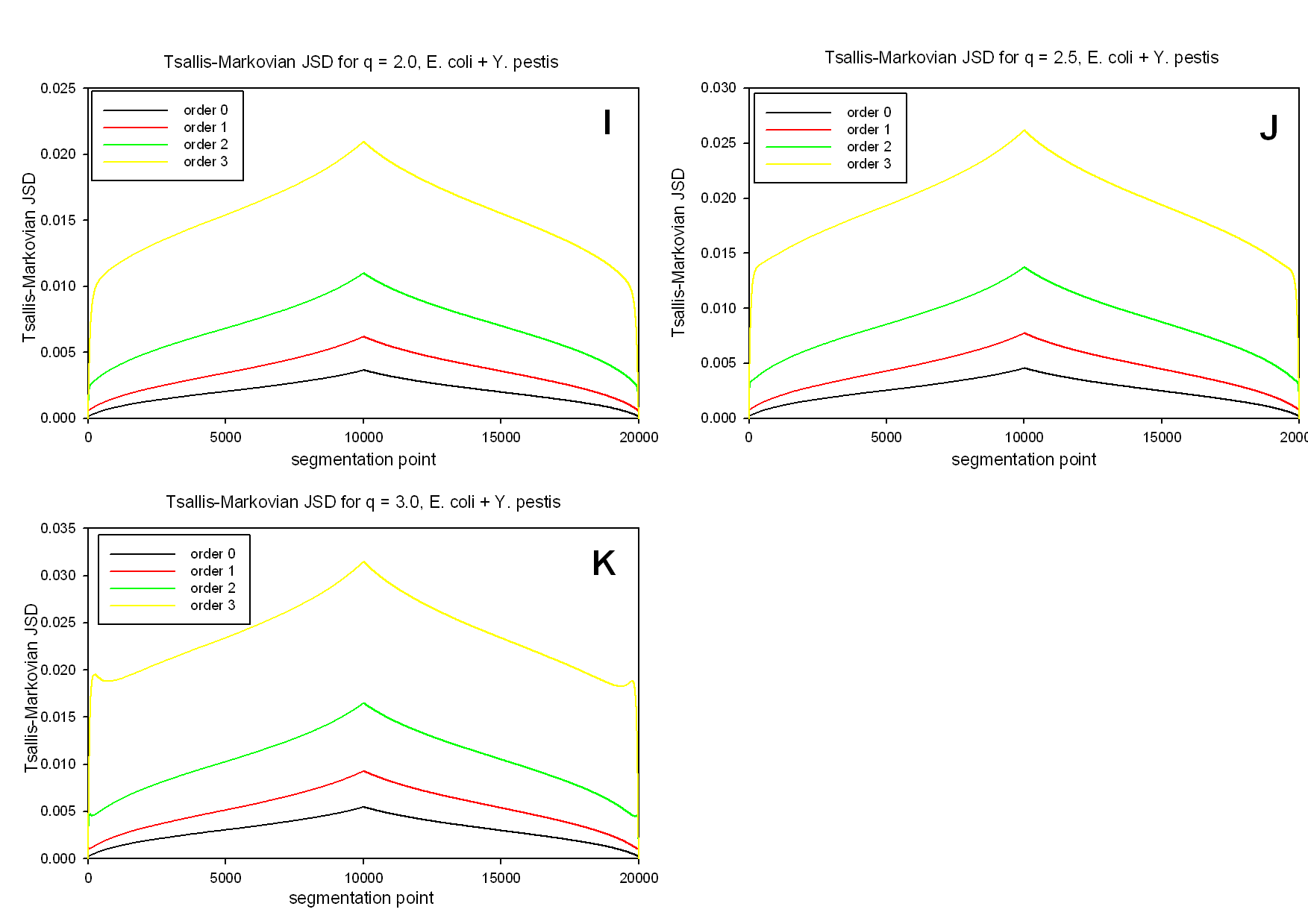

Supplement: Figure S6 — As in Figure S5, but for Tsallis statistics’ parameter q = 2.0, 2.5, 3.0. (TIF) [file pone.0093532.s006.tif]

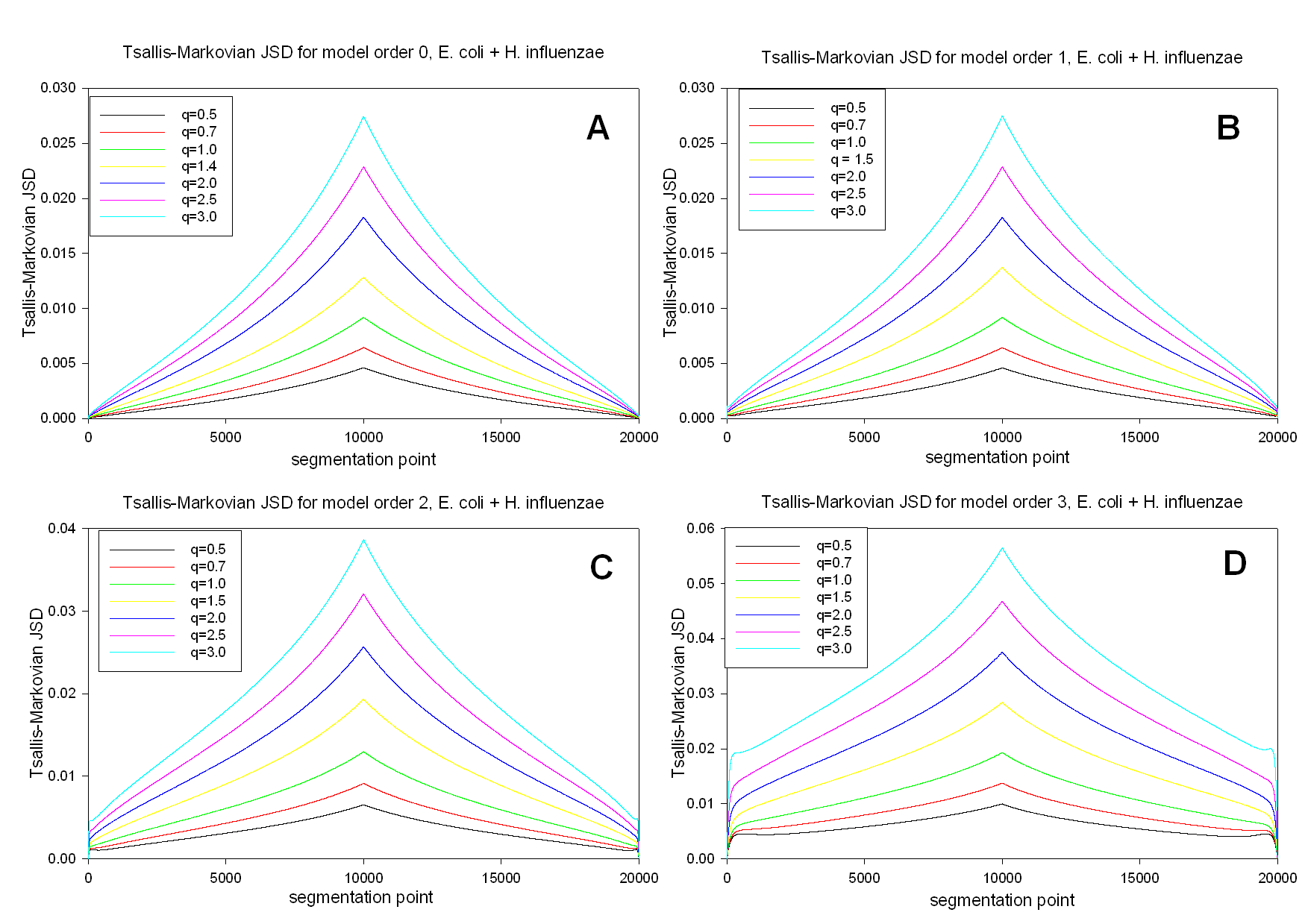

Supplement: Figure S7 — Mean values of non-extensive MJSD at each position of the chimeric sequence constructs E. coli H. influenzae, for model order m = 0–3. For each model order, plots are shown for different values of Tsallis statistics’ parameter q, in the range 0.5–3. The chimeric constructs of size 20 Kbp are comprised of two equal sized sequences, with each component sequence of length 10 Kbp obtained from the genome of each organism. (TIF) [file pone.0093532.s007.tif]

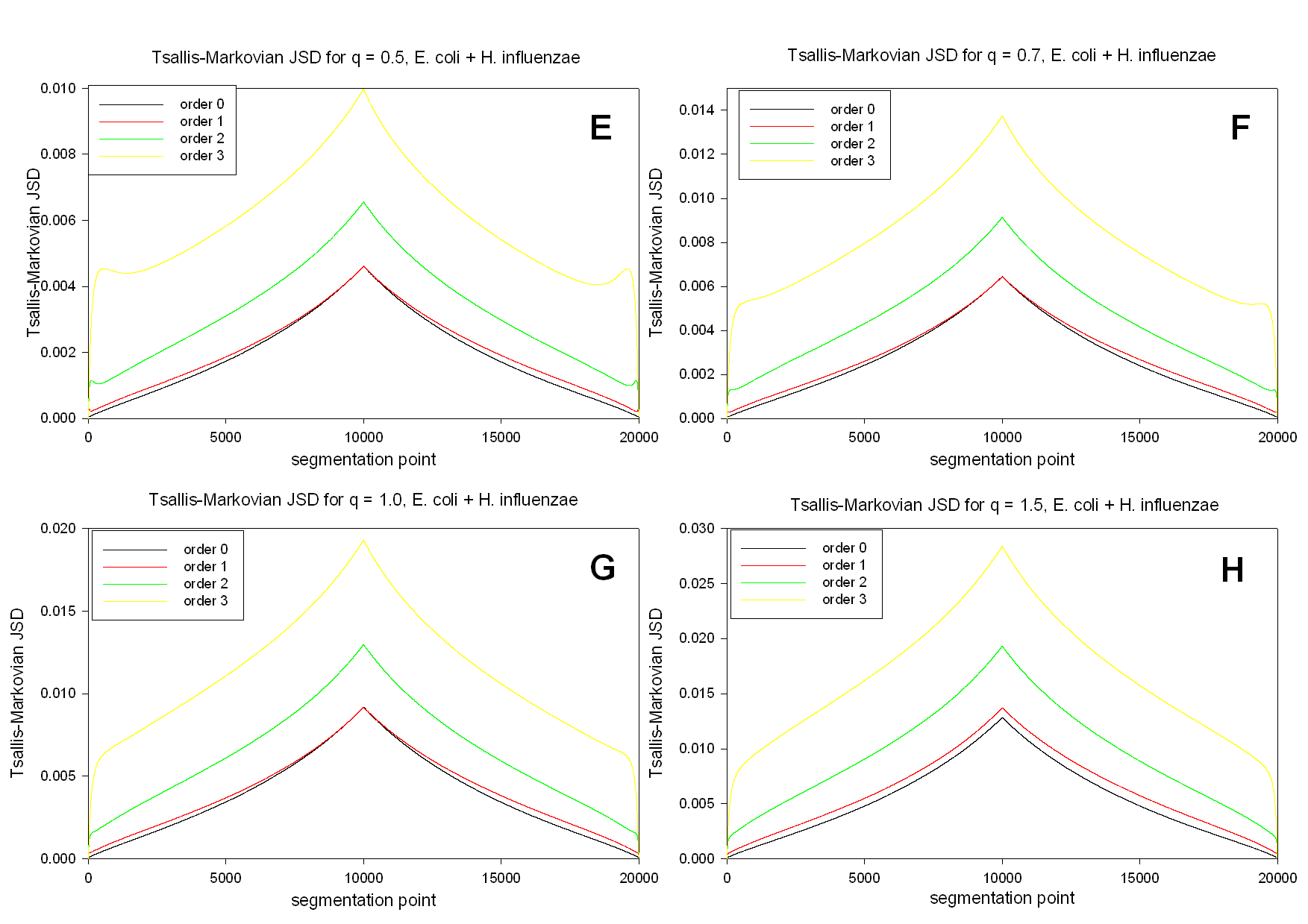

Supplement: Figure S8 — Mean values of non-extensive MJSD at each position of the chimeric sequence constructs E. coli H. influenzae, for Tsallis statistics’ parameter q = 0.5, 0.7, 1.0, 1.5. For each q, plots are shown for different model orders, in the range 0–3. The chimeric constructs of size 20 Kbp are comprised of two equal sized sequences, with each component sequence of length 10 Kbp obtained from the genome of each organism. (TIF) [file pone.0093532.s008.tif]

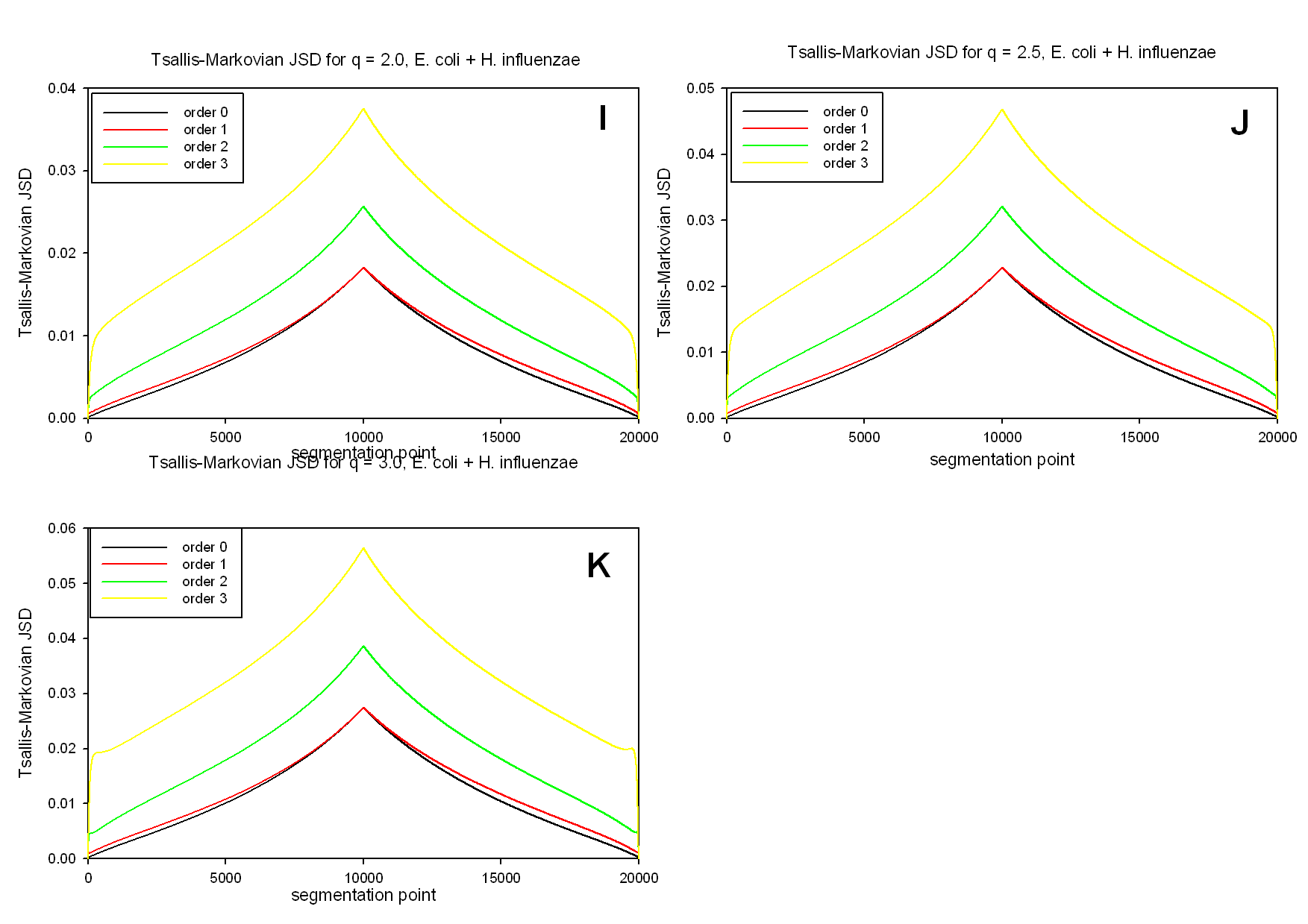

Supplement: Figure S9 — As in Figure S8, but for Tsallis statistics’ parameter q = 2.0, 2.5, 3.0. (TIF) [file pone.0093532.s009.tif]

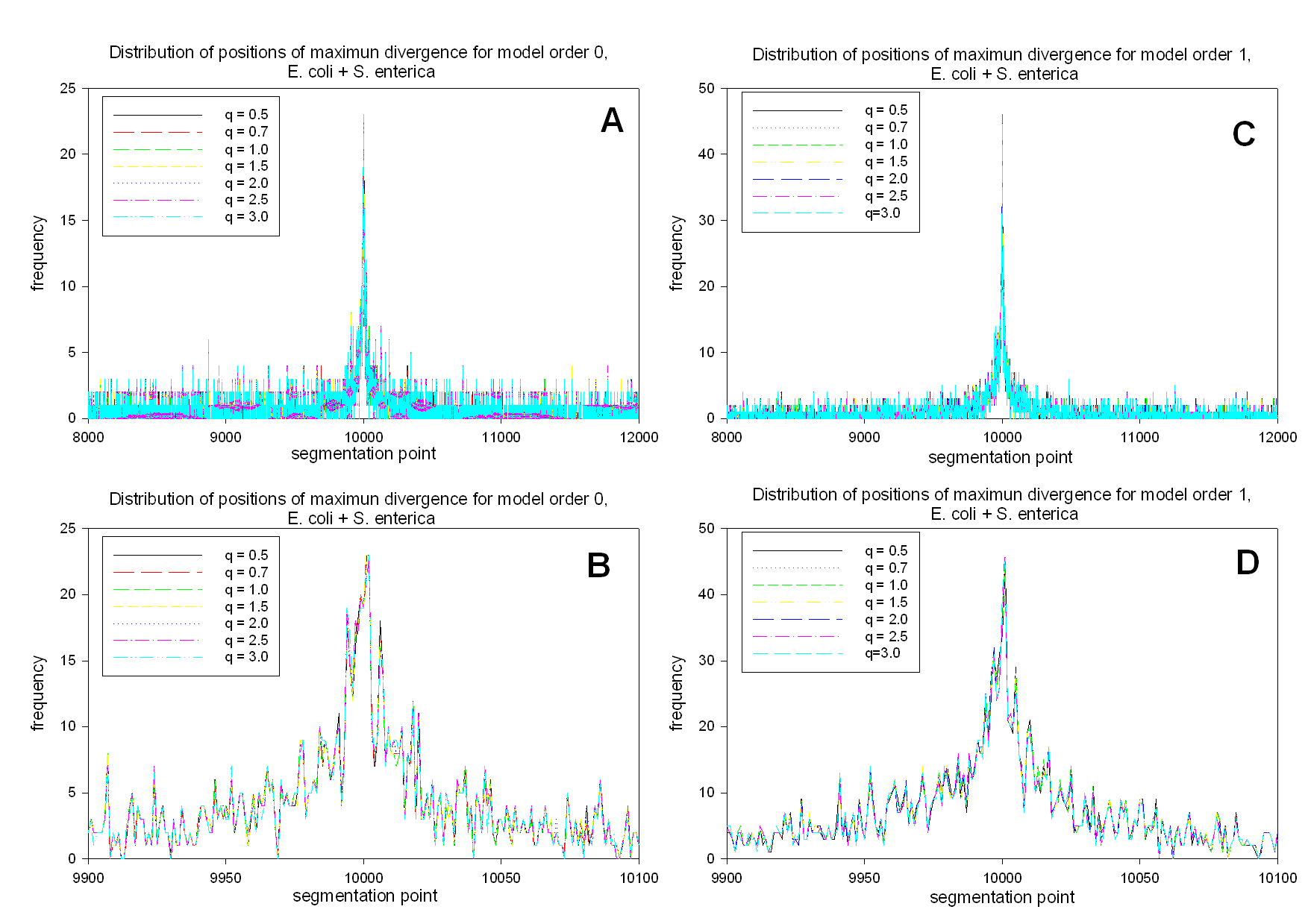

Supplement: Figure S10 — Frequency distribution of position with maximum value of non-extensive MJSD for the chimeric sequence constructs E. coli S. enterica, for model order m = 0 (A, B) and 1 (C, D). For each model order, distributions are shown for different values of Tsallis statistics’ parameter q, in the range 0.5–3. The chimeric constructs of size 20 Kbp are comprised of two equal sized sequences, with each component sequence of length 10 Kbp obtained from the genome of each organism. (TIF) [file pone.0093532.s010.tif]

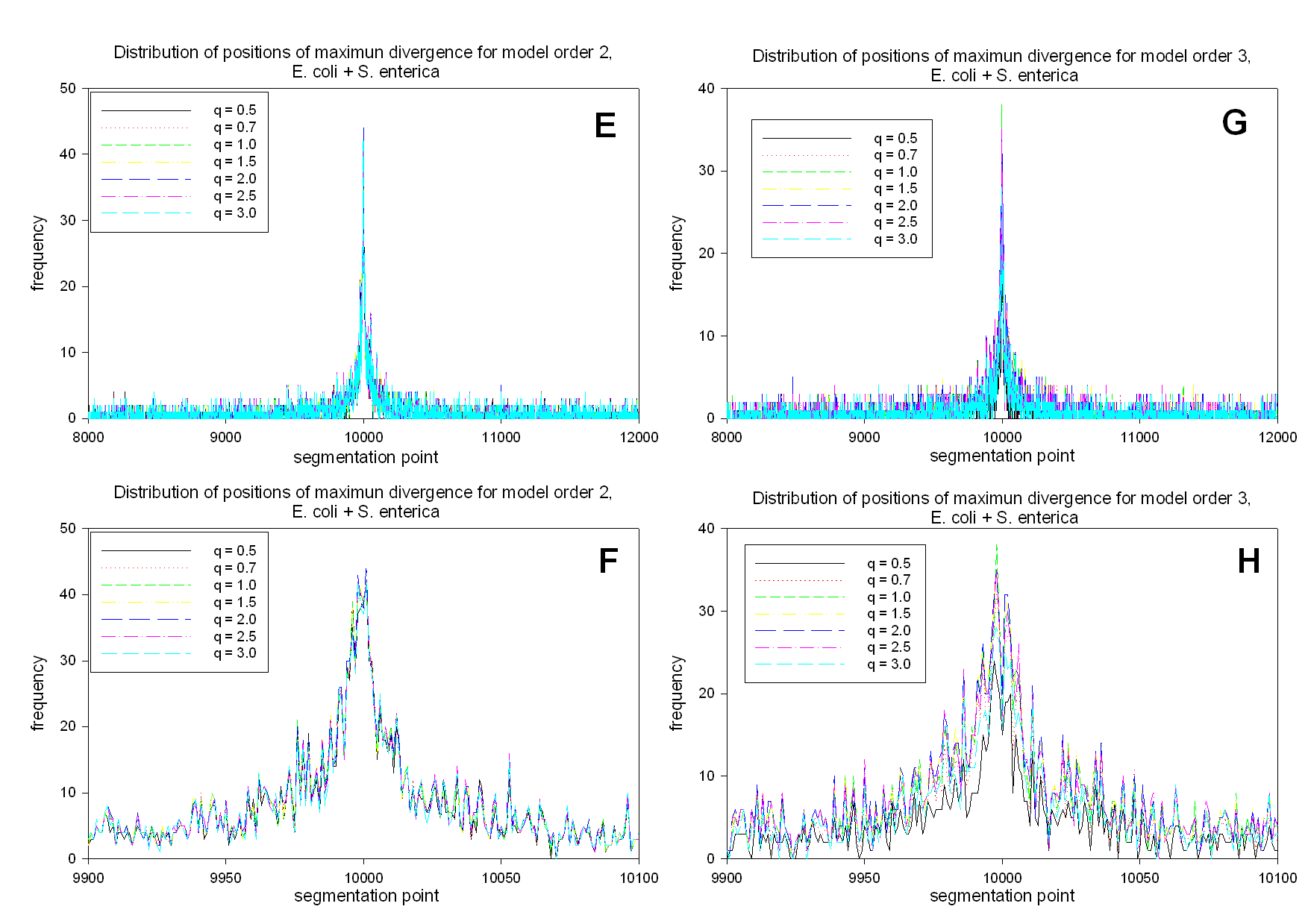

Supplement: Figure S11 — As in Figure S10, but for model order m = 2 (E, F) and 3 (G, H). (TIF) [file pone.0093532.s011.tif]

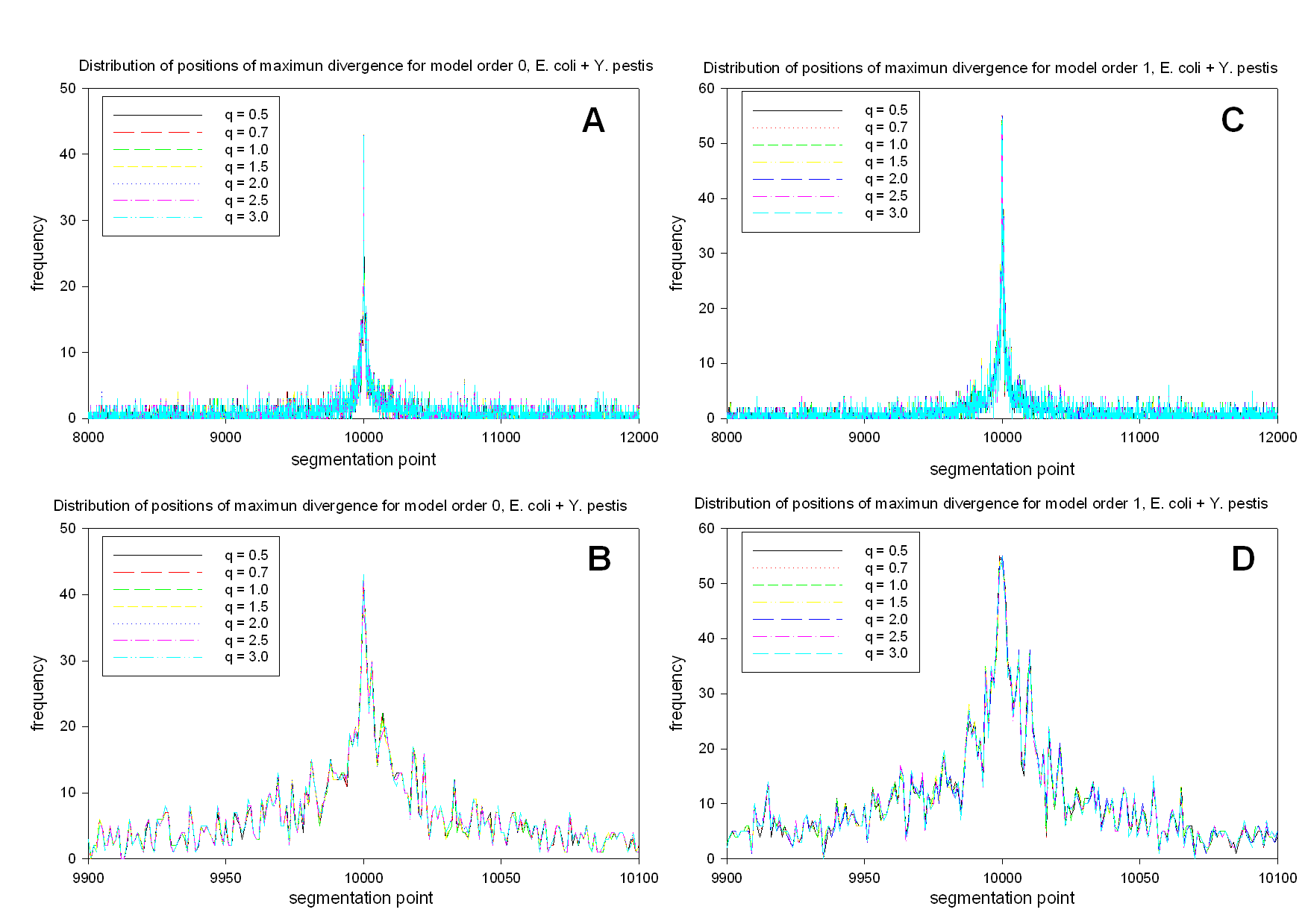

Supplement: Figure S12 — Frequency distribution of position with maximum value of non-extensive MJSD for the chimeric sequence constructs E. coli Y. pestis, for model order m = 0 (A, B) and 1 (C, D). For each model order, distributions are shown for different values of Tsallis statistics’ parameter q, in the range 0.5–3. The chimeric constructs of size 20 Kbp are comprised of two equal sized sequences, with each component sequence of length 10 Kbp obtained from the genome of each organism. (TIF) [file pone.0093532.s012.tif]

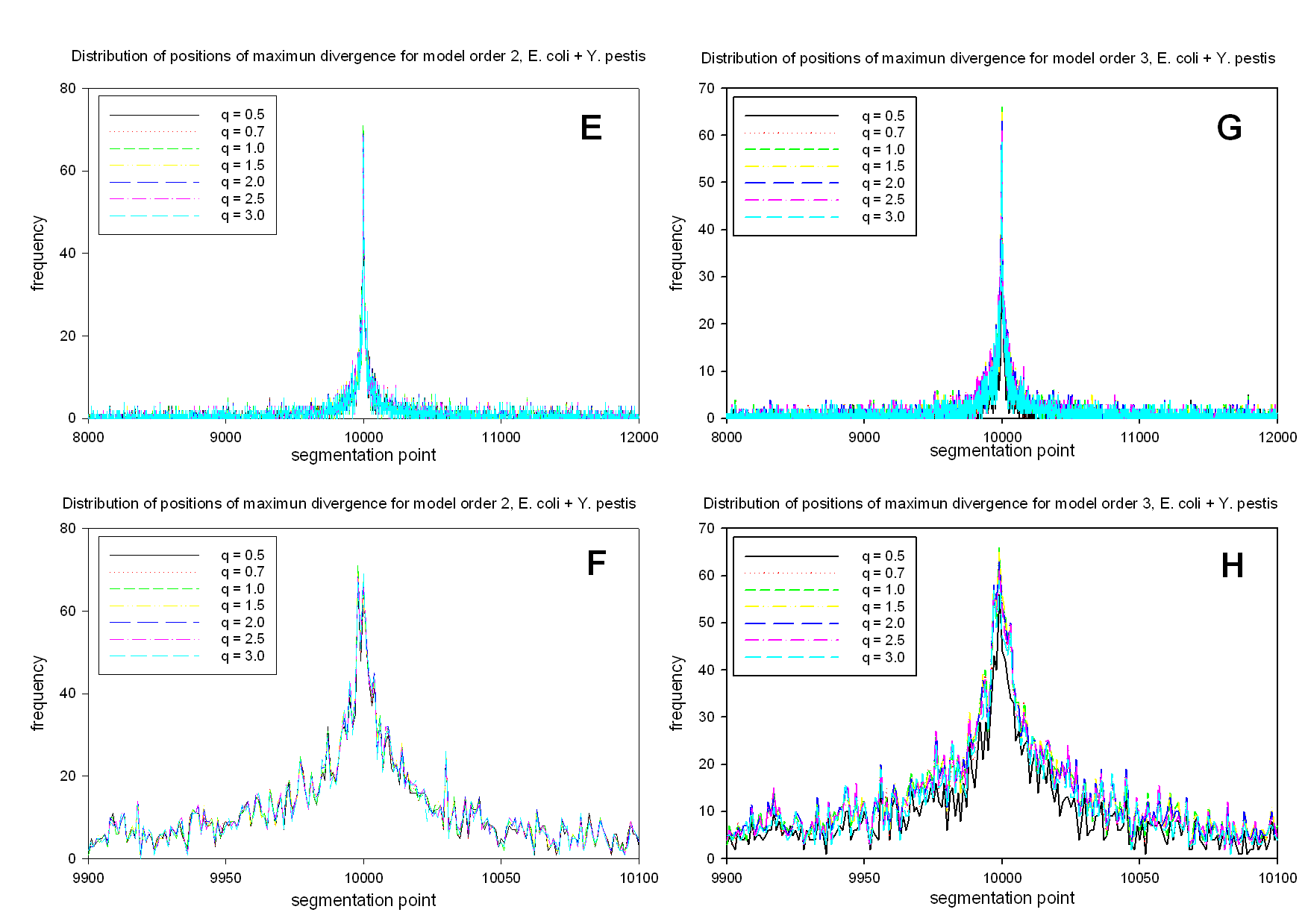

Supplement: Figure S13 — As in Figure S12, but for model order m = 2 (E, F) and 3 (G, H). (TIF) [file pone.0093532.s013.tif]

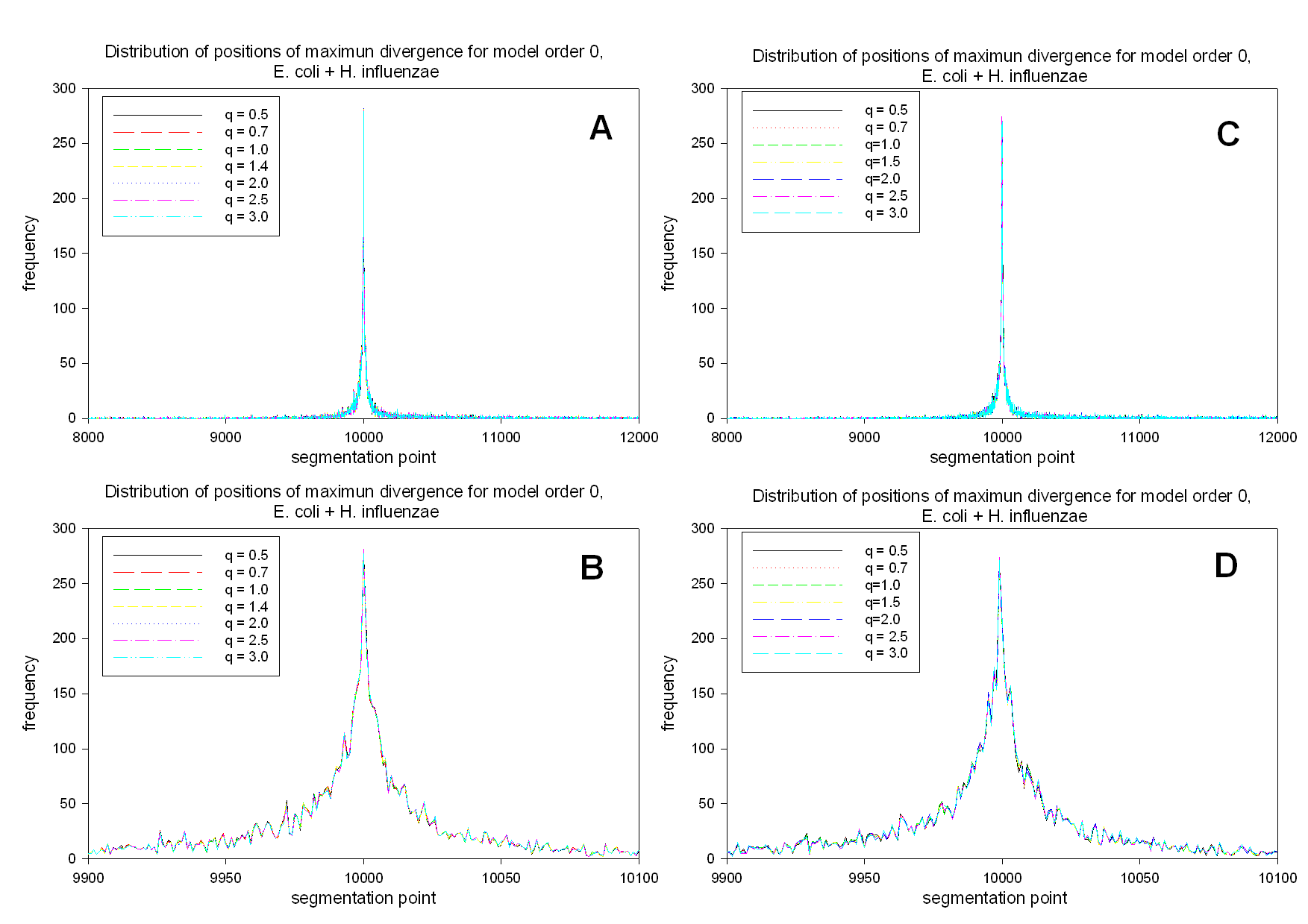

Supplement: Figure S14 — Frequency distribution of position with maximum value of non-extensive MJSD for the chimeric sequence constructs E. coli H. influenzae, for model order m = 0 (A, B) and 1 (C, D). For each model order, distributions are shown for different values of Tsallis statistics’ parameter q, in the range 0.5–3. The chimeric constructs of size 20 Kbp are comprised of two equal sized sequences, with each component sequence of length 10 Kbp obtained from the genome of each organism. (TIF) [file pone.0093532.s014.tif]

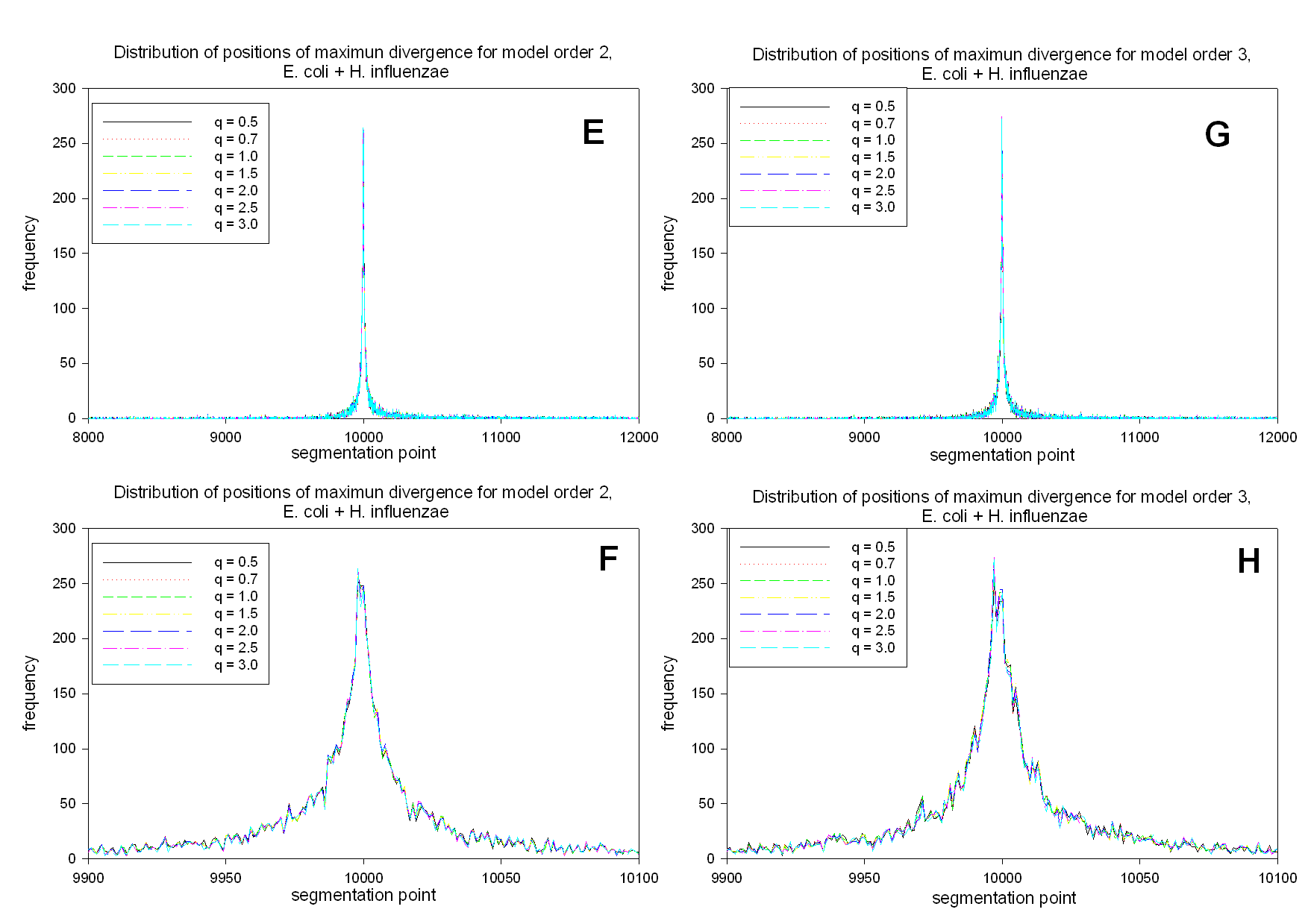

Supplement: Figure S15 — As in Figure S14, but for model order m = 2 (E, F) and 3 (G, H). (TIF) [file pone.0093532.s015.tif]

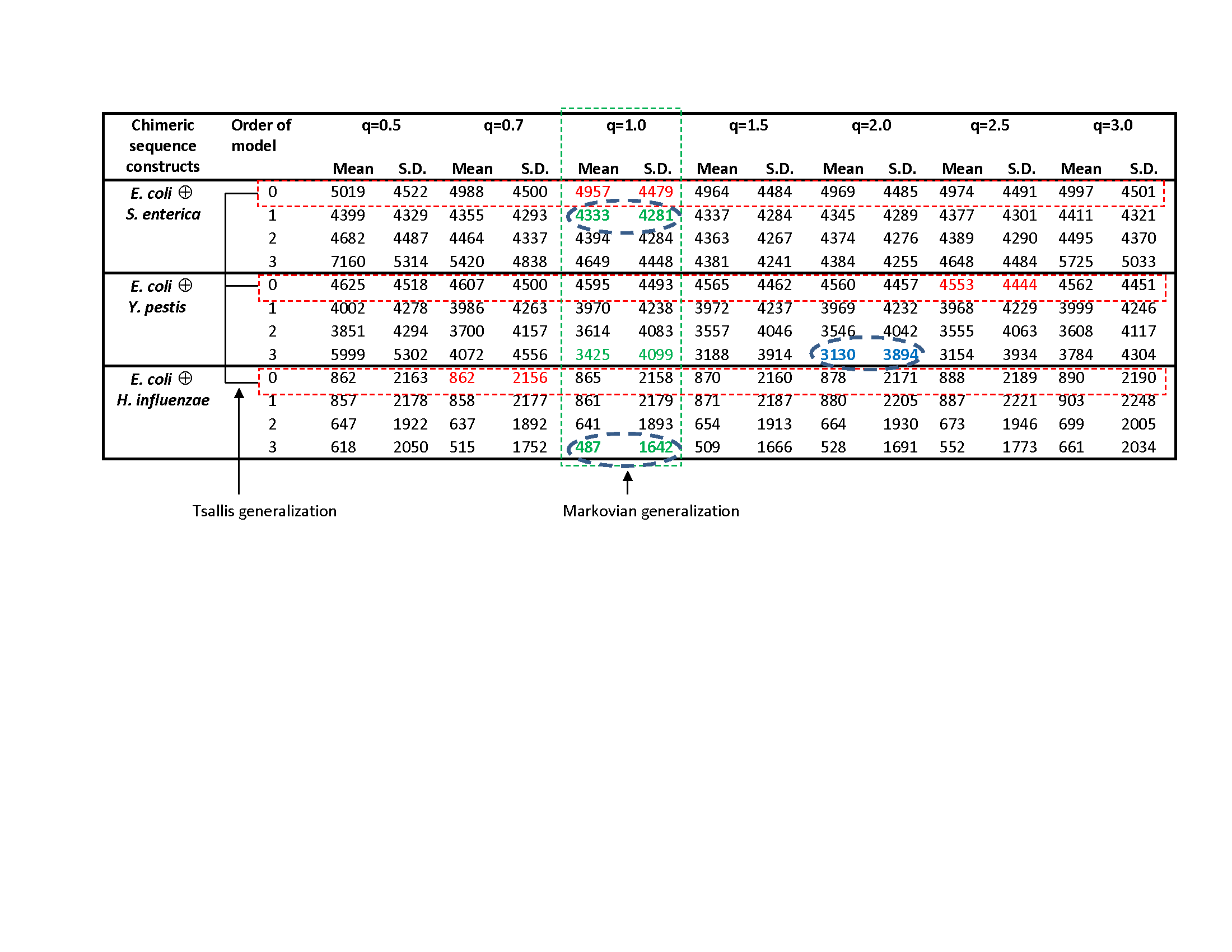

Supplement: Figure S16 — Error (in base pairs) in detecting the join point in the chimeric sequence constructs for E. coli S. enterica, E. coli Y. pestis, and E. coli H. influenzae (denotes concatenation). The proposed Tsallis-Markovian generalization of the Jensen-Shannon divergence measure was used to obtain the mean and standard deviation of the error from 5,000 replicates for each type of chimeric sequence constructs. The error in localizing the join point was obtained as the absolute difference between the position where the divergence was maximized and the position of the join point (at 5 Kbp) in a chimeric sequence construct of size 20 Kbp (5 Kbp sequence from non-E. coli organism concatenated with 15 Kbp from E. coli). Error statistics for the two special cases of the proposed generalized measure is shown within rectangular boxes– the Markovian generalization (q = 1) in dashed green border box and Tsallis non-extensive generalization (model order = 0) in dashed red border boxes. The minimum values of mean and standard deviation of the error for each chimeric construct type are shown encircled and bold faced. (TIFF) [file pone.0093532.s016.tif]

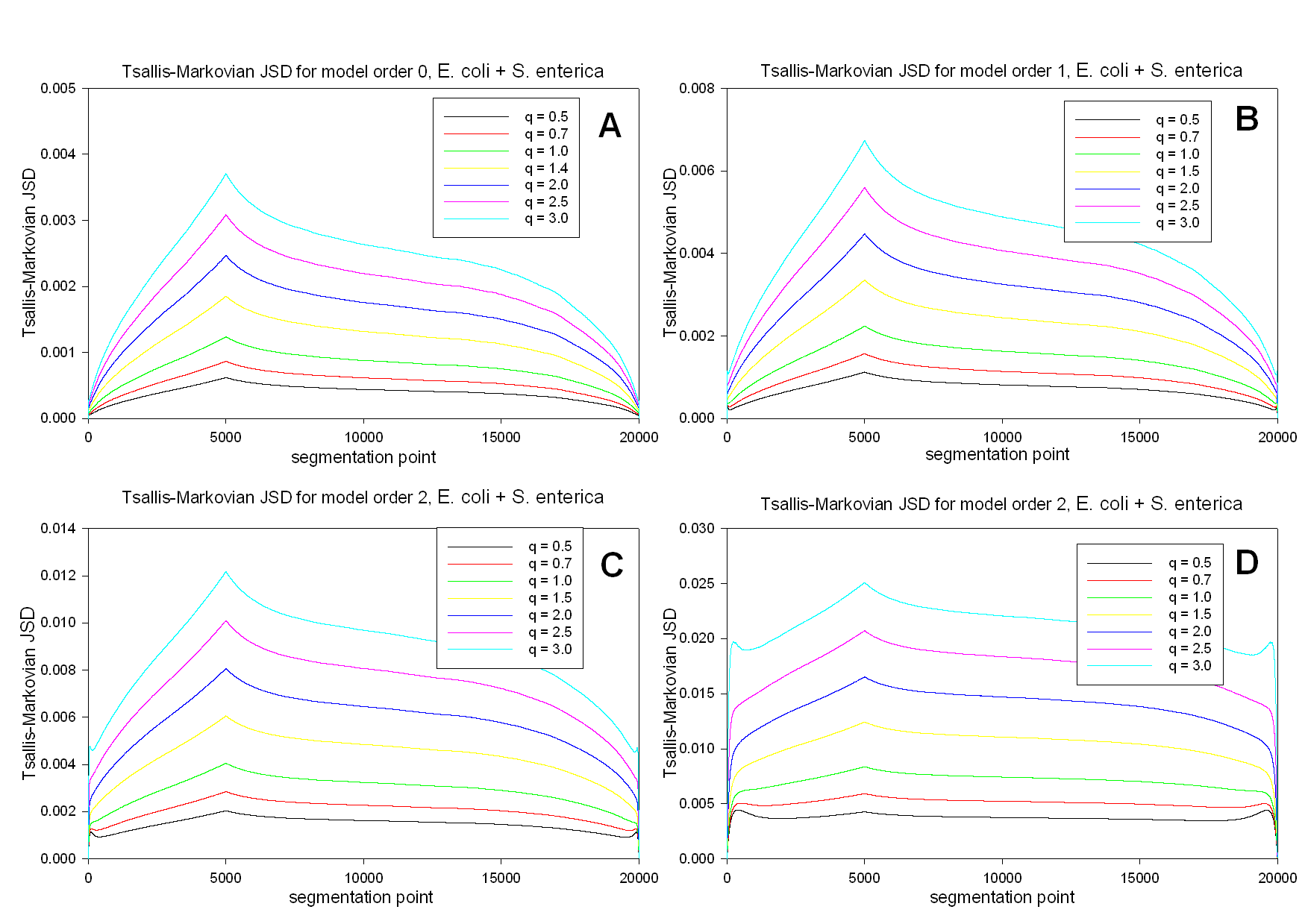

Supplement: Figure S17 — Mean values of non-extensive MJSD at each position of the chimeric sequence constructs E. coli S. enterica, for model order m = 0–3. For each model order, plots are shown for different values of Tsallis statistics’ parameter q, in the range 0.5–3. The chimeric constructs of size 20 Kbp are comprised of two sequences, one component sequence of length 5 Kbp obtained from the genome of S. enterica and the other of length 15 Kbp from the genome of E. coli. (TIF) [file pone.0093532.s017.tif]

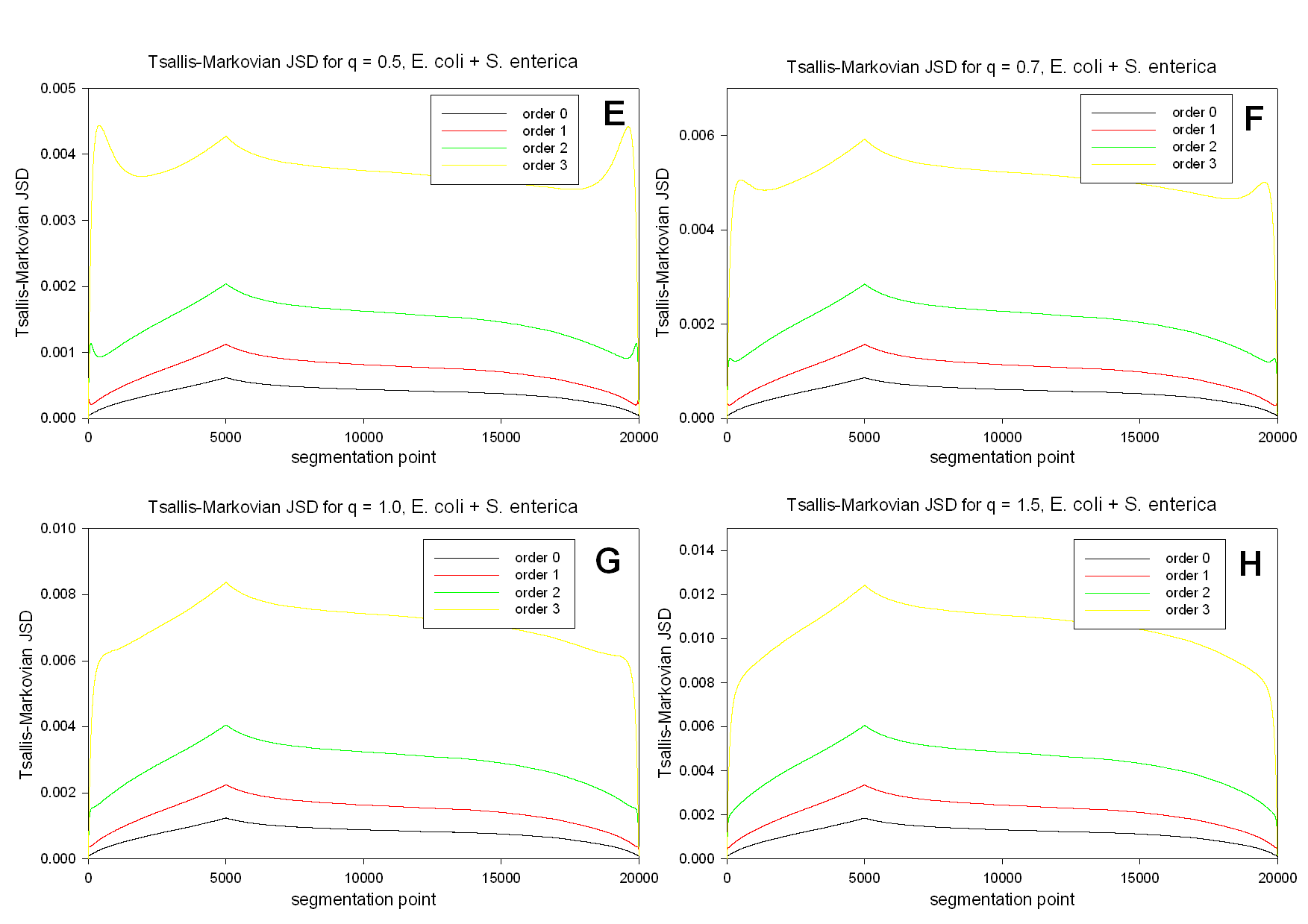

Supplement: Figure S18 — Mean values of non-extensive MJSD at each position of the chimeric sequence constructs E. coli S. enterica, for Tsallis statistics’ parameter q = 0.5, 0.7, 1.0, 1.5. For each q, plots are shown for different model orders, in the range 0–3. The chimeric constructs of size 20 Kbp are comprised of two sequences, one component sequence of length 5 Kbp obtained from the genome of S. enterica and the other of length 15 Kbp from the genome of E. coli. (TIF) [file pone.0093532.s018.tif]

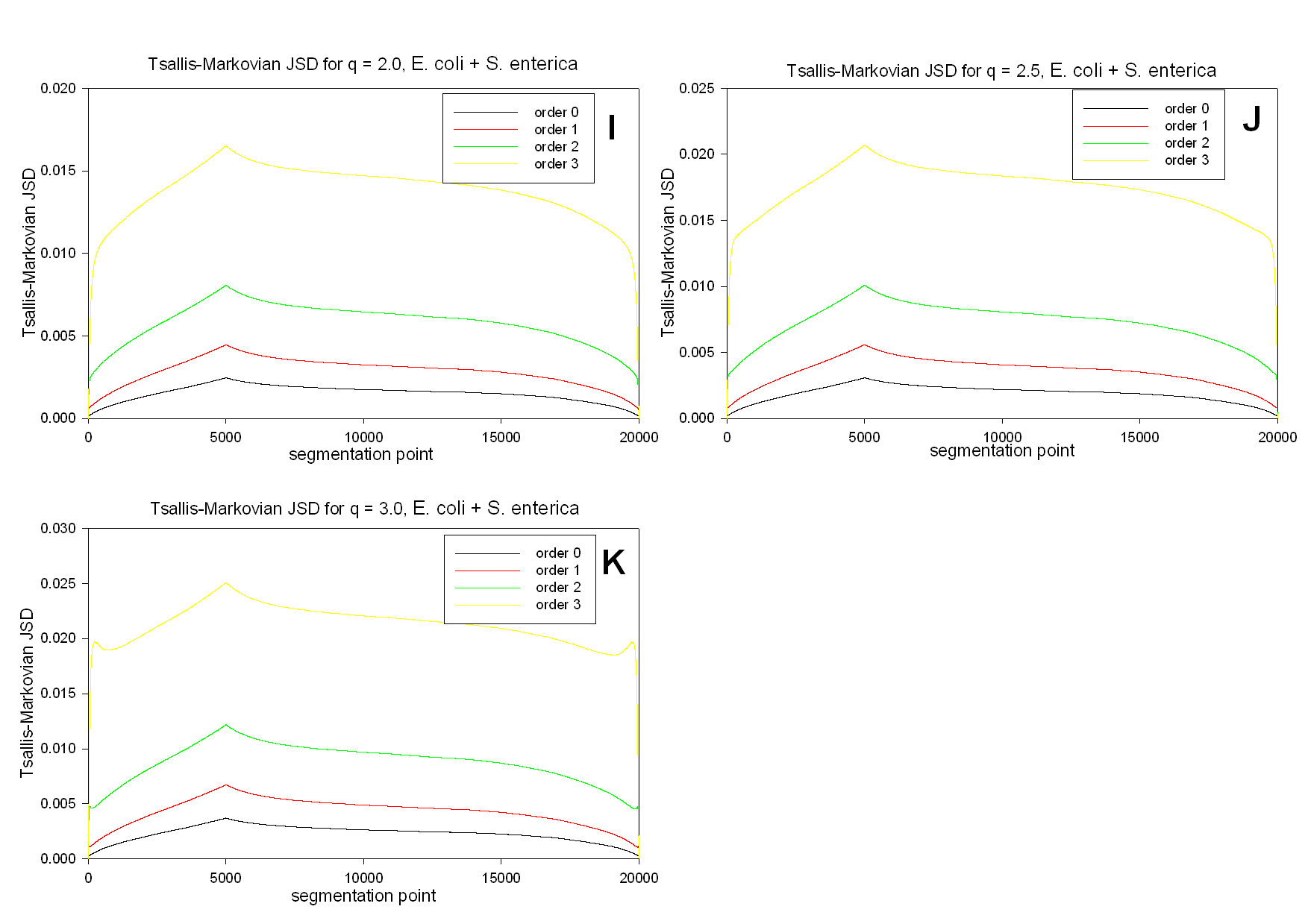

Supplement: Figure S19 — As in Figure S18, but for Tsallis statistics’ parameter q = 2.0, 2.5, 3.0. (TIF) [file pone.0093532.s019.tif]

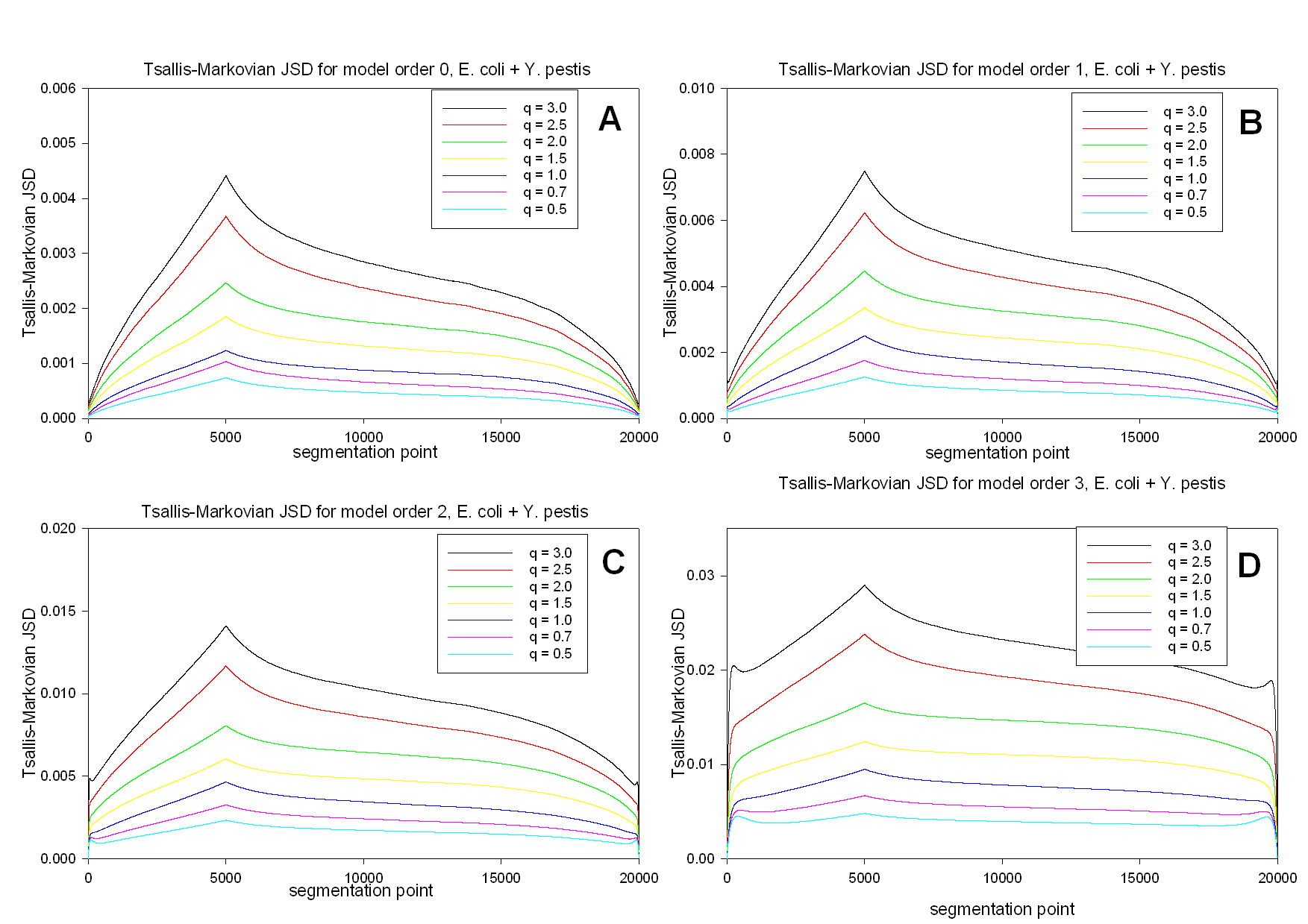

Supplement: Figure S20 — Mean values of non-extensive MJSD at each position of the chimeric sequence constructs E. coli Y. pestis, for model order m = 0–3. For each model order, plots are shown for different values of Tsallis statistics’ parameter q, in the range 0.5–3. The chimeric constructs of size 20 Kbp are comprised of two sequences, one component sequence of length 5 Kbp obtained from the genome of Y. pestis and the other of length 15 Kbp from the genome of E. coli. (TIF) [file pone.0093532.s020.tif]

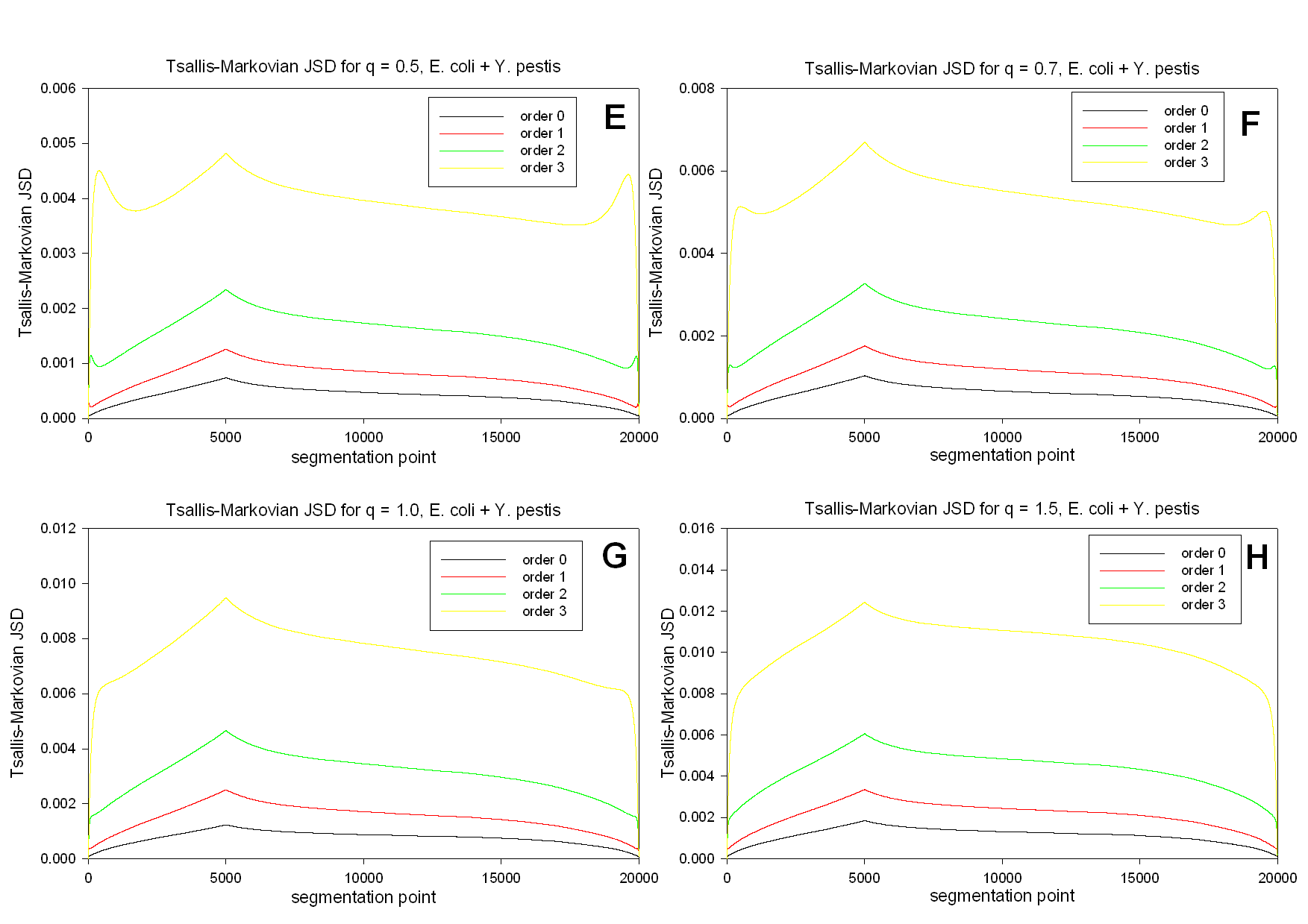

Supplement: Figure S21 — Mean values of non-extensive MJSD at each position of the chimeric sequence constructs E. coli Y. pestis, for Tsallis statistics’ parameter q = 0.5, 0.7, 1.0, 1.5. For each q, plots are shown for different model orders, in the range 0–3. The chimeric constructs of size 20 Kbp are comprised of two sequences, one component sequence of length 5 Kbp obtained from the genome of Y. pestis and the other of length 15 Kbp from the genome of E. coli. (TIF) [file pone.0093532.s021.tif]

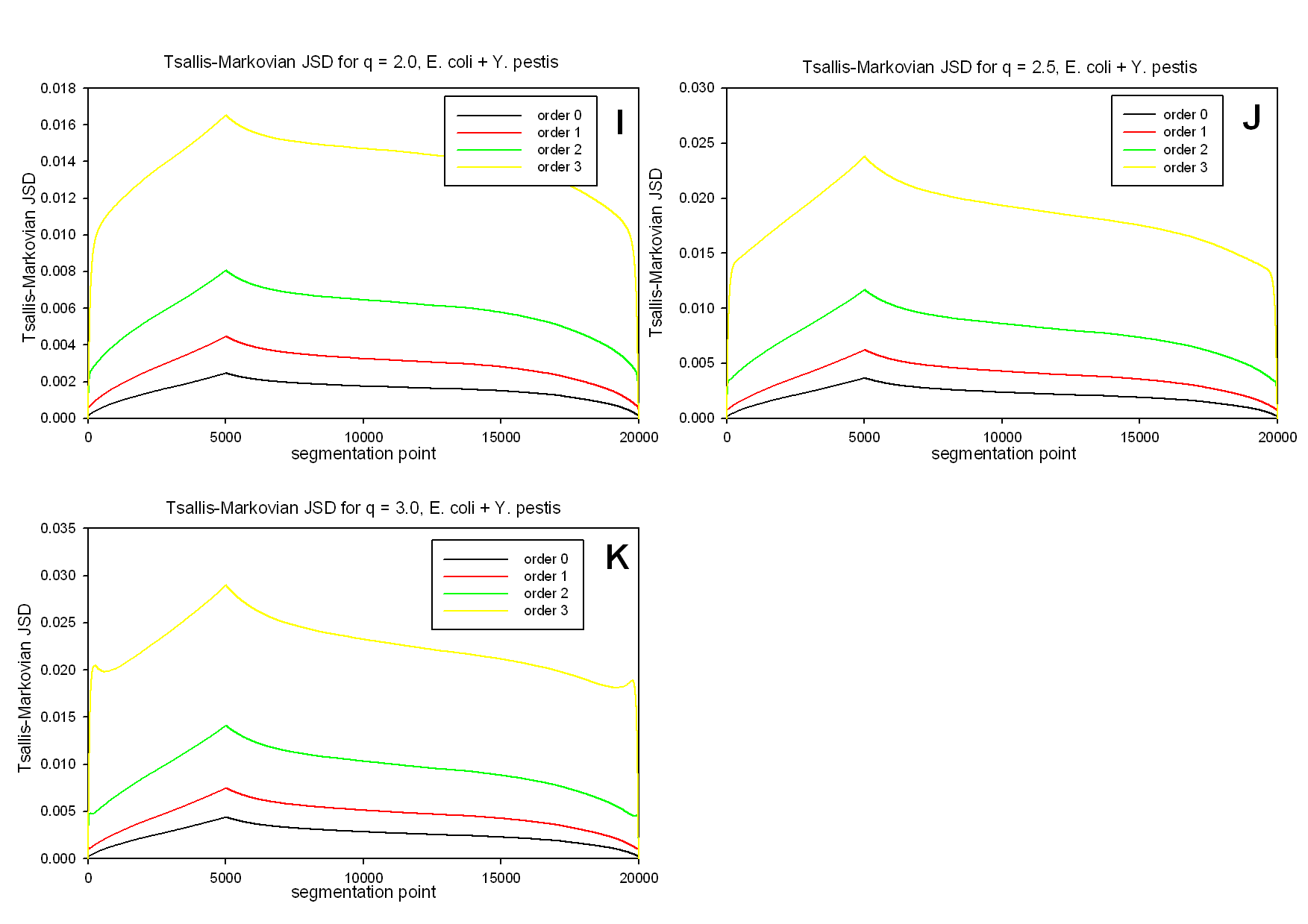

Supplement: Figure S22 — As in Figure S21, but for Tsallis statistics’ parameter q = 2.0, 2.5, 3.0. (TIF) [file pone.0093532.s022.tif]

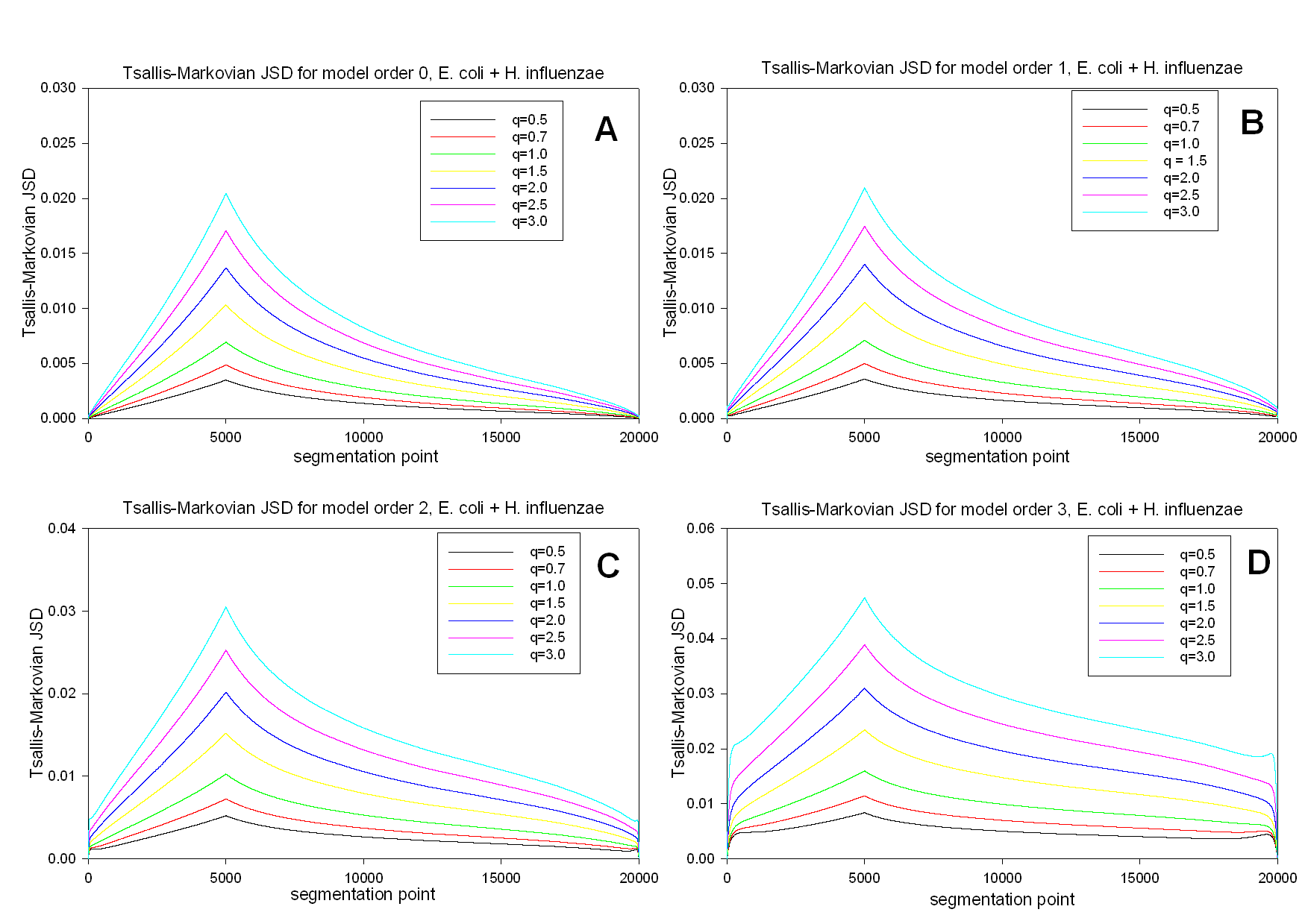

Supplement: Figure S23 — Mean values of non-extensive MJSD at each position of the chimeric sequence constructs E. coli H. influenzae, for model order m = 0–3. For each model order, plots are shown for different values of Tsallis statistics’ parameter q, in the range 0.5–3. The chimeric constructs of size 20 Kbp are comprised of two sequences, one component sequence of length 5 Kbp obtained from the genome of H. influenzae and the other of length 15 Kbp from the genome of E. coli. (TIF) [file pone.0093532.s023.tif]

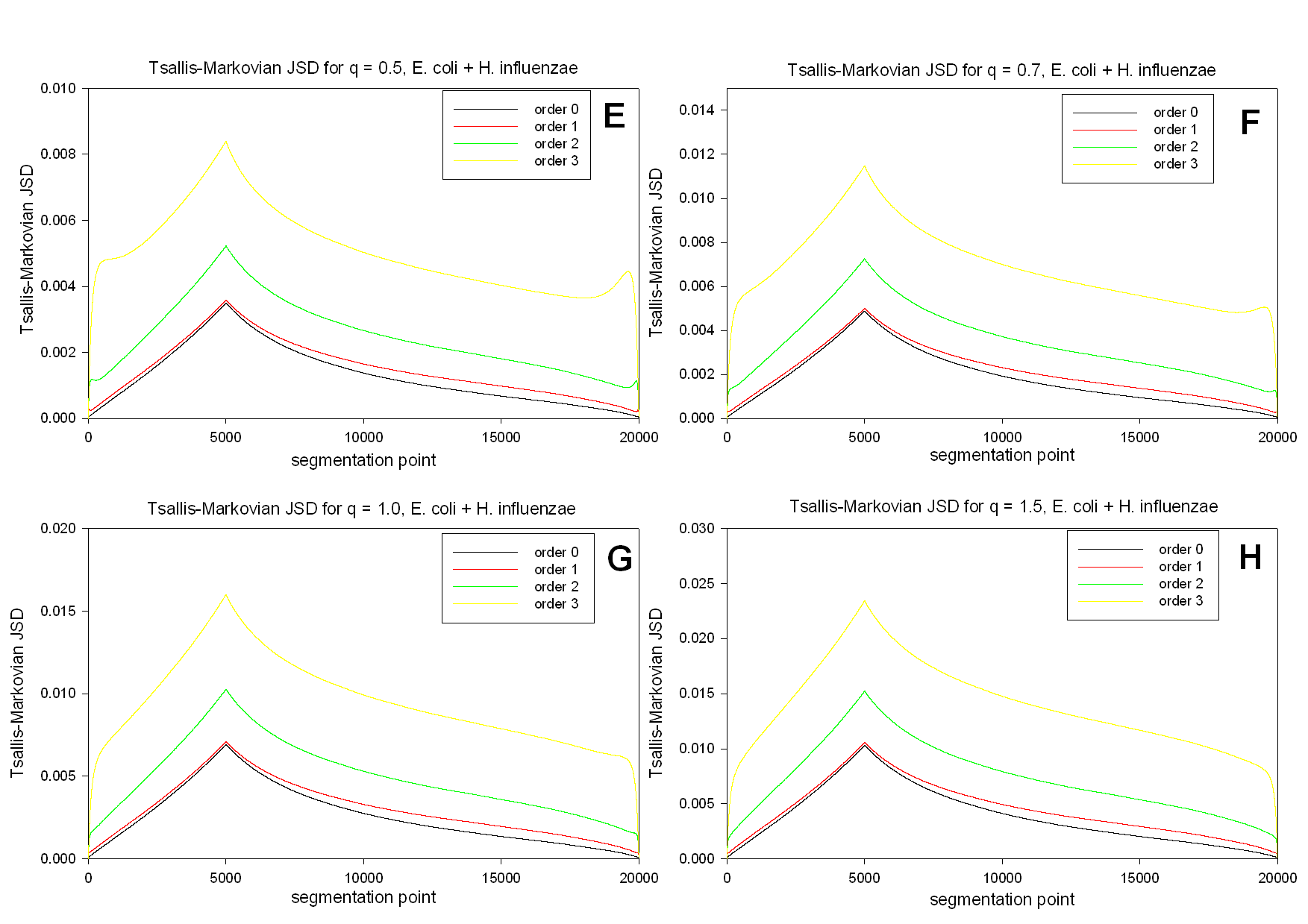

Supplement: Figure S24 — Mean values of non-extensive MJSD at each position of the chimeric sequence constructs E. coli H. influenzae, for Tsallis statistics’ parameter q = 0.5, 0.7, 1.0, 1.5. For each q, plots are shown for different model orders, in the range 0–3. The chimeric constructs of size 20 Kbp are comprised of two sequences, one component sequence of length 5 Kbp obtained from the genome of H. influenzae and the other of length 15 Kbp from the genome of E. coli. (TIF) [file pone.0093532.s024.tif]

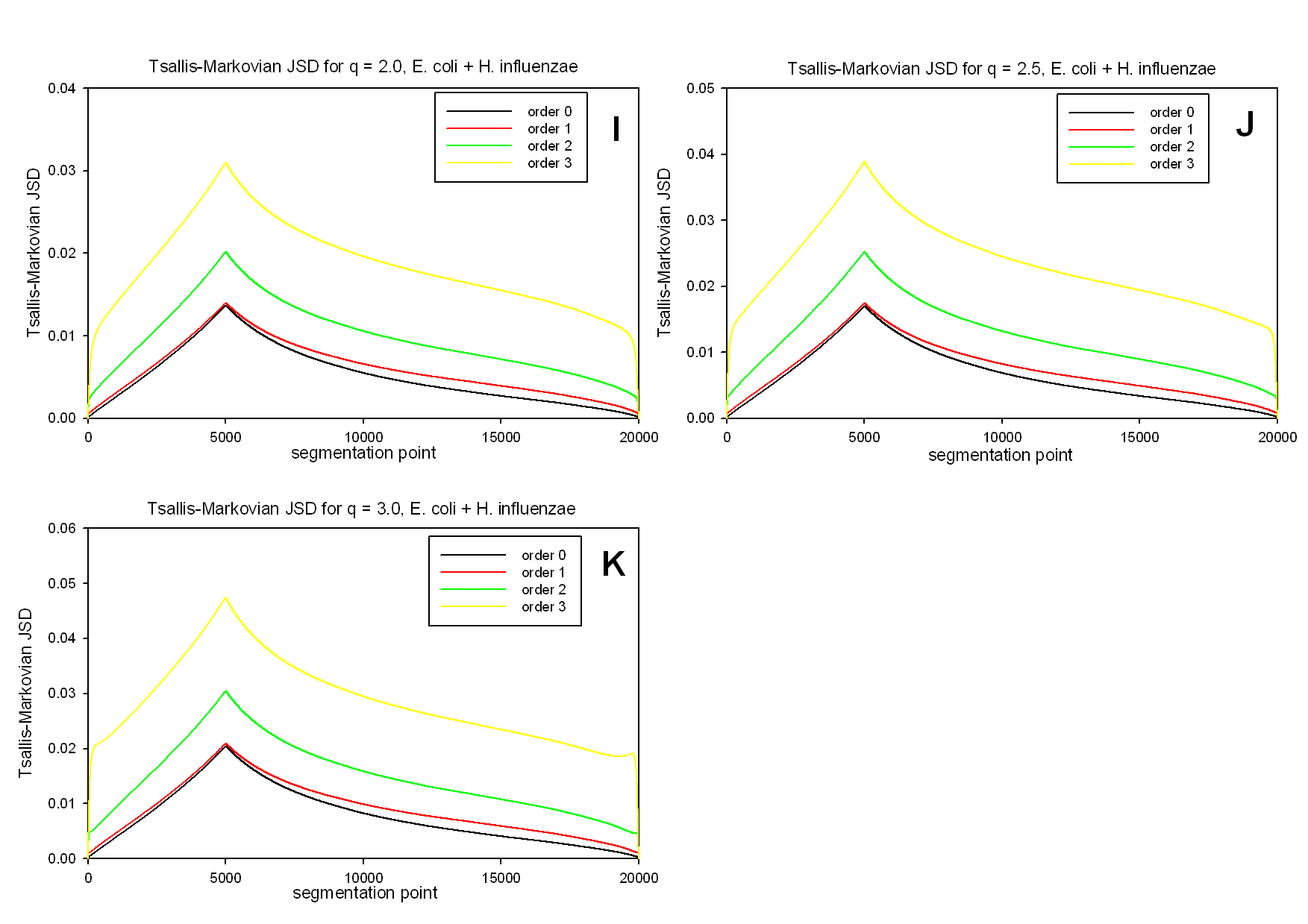

Supplement: Figure S25 — As in Figure S24, but for Tsallis statistics’ parameter q = 2.0, 2.5, 3.0. (TIF) [file pone.0093532.s025.tif]

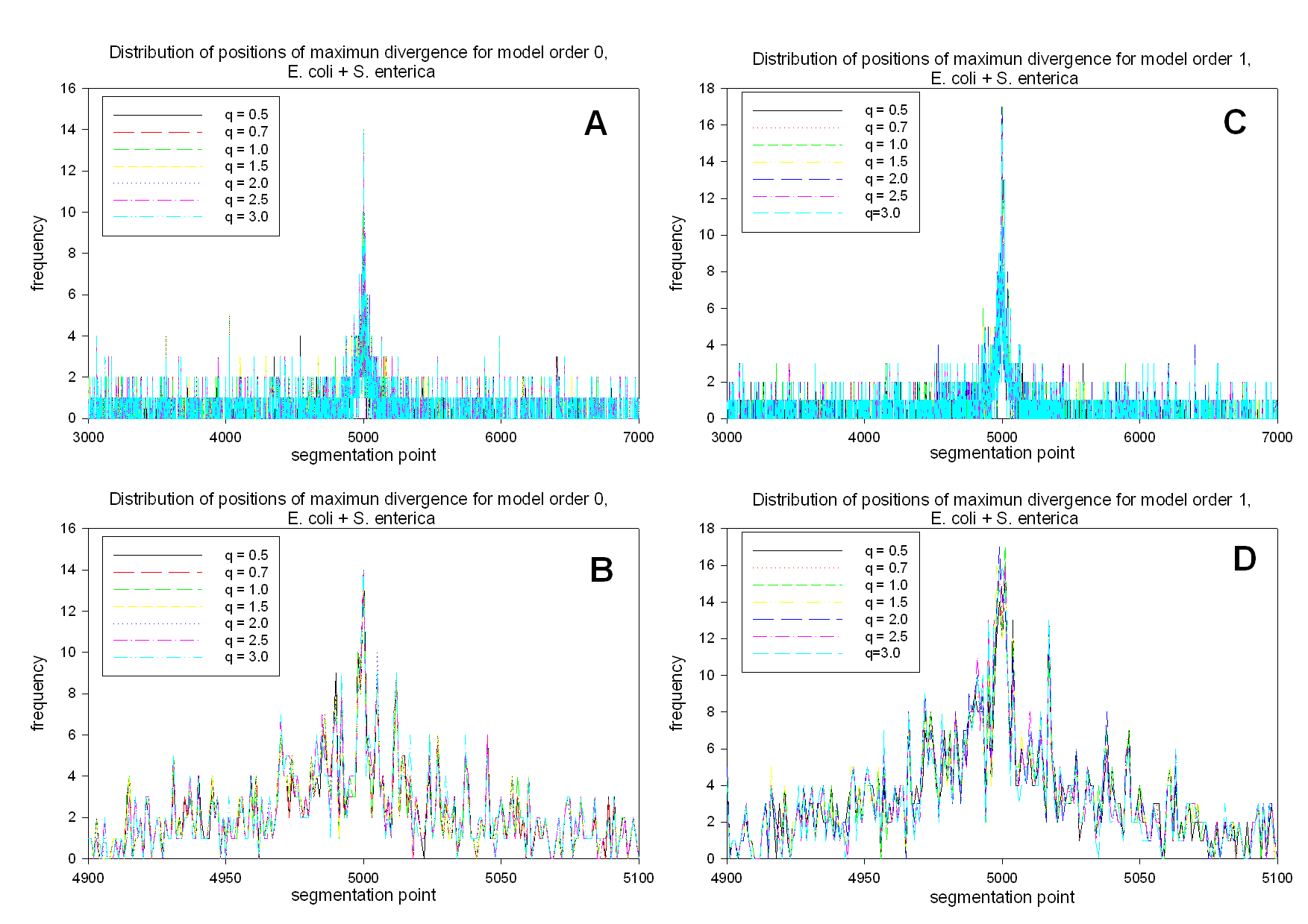

Supplement: Figure S26 — Frequency distribution of position with maximum value of non-extensive MJSD for the chimeric sequence constructs E. coli S. enterica, for model order m = 0 (A, B) and 1 (C, D). For each model order, distributions are shown for different values of Tsallis statistics’ parameter q, in the range 0.5–3. The chimeric constructs of size 20 Kbp are comprised of two sequences, one component sequence of length 5 Kbp obtained from the genome of S. enterica and the other of length 15 Kbp from the genome of E. coli. (TIF) [file pone.0093532.s026.tif]

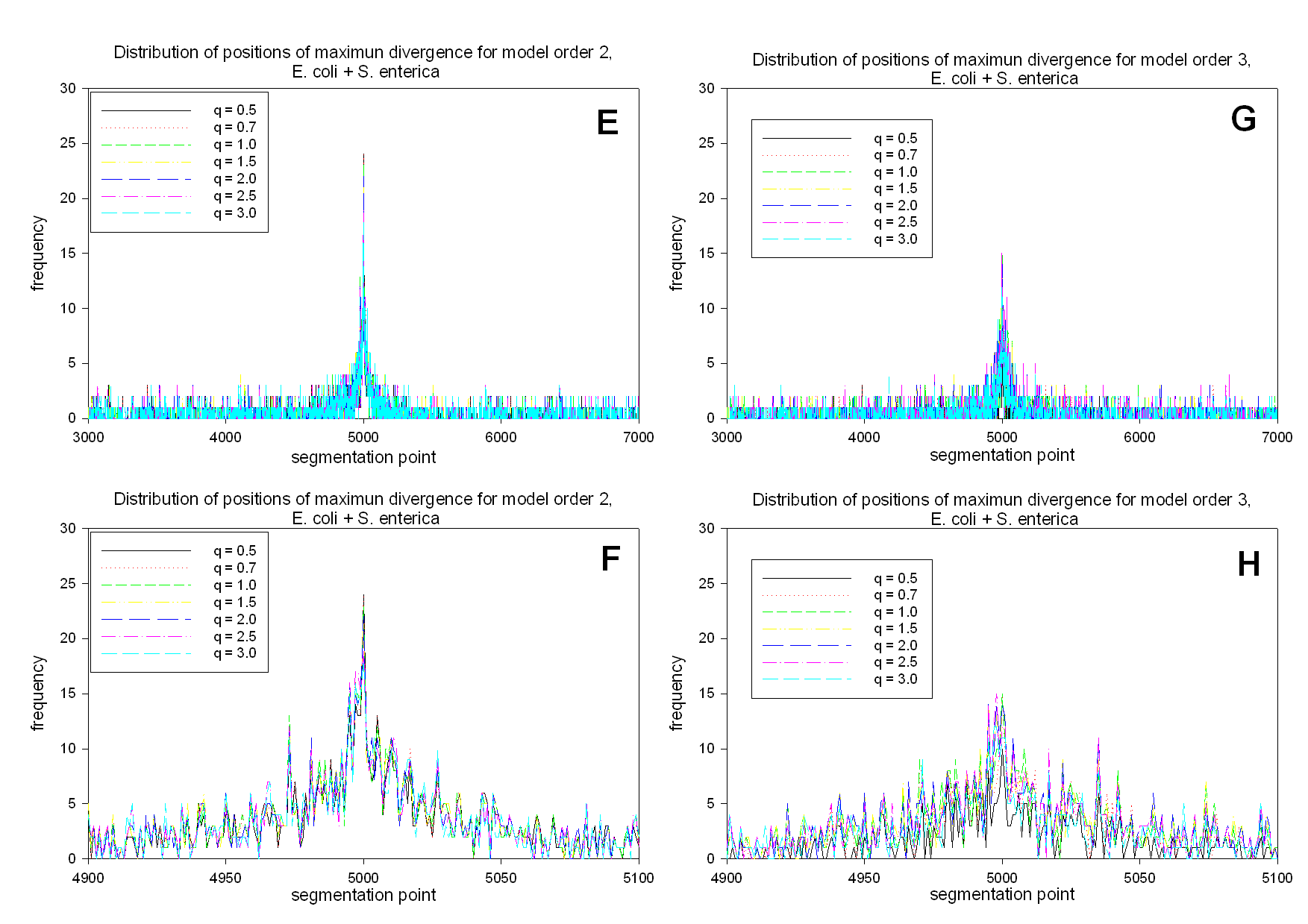

Supplement: Figure S27 — As in Figure S26, but for model order m = 2 (E, F) and 3 (G, H). (TIF) [file pone.0093532.s027.tif]

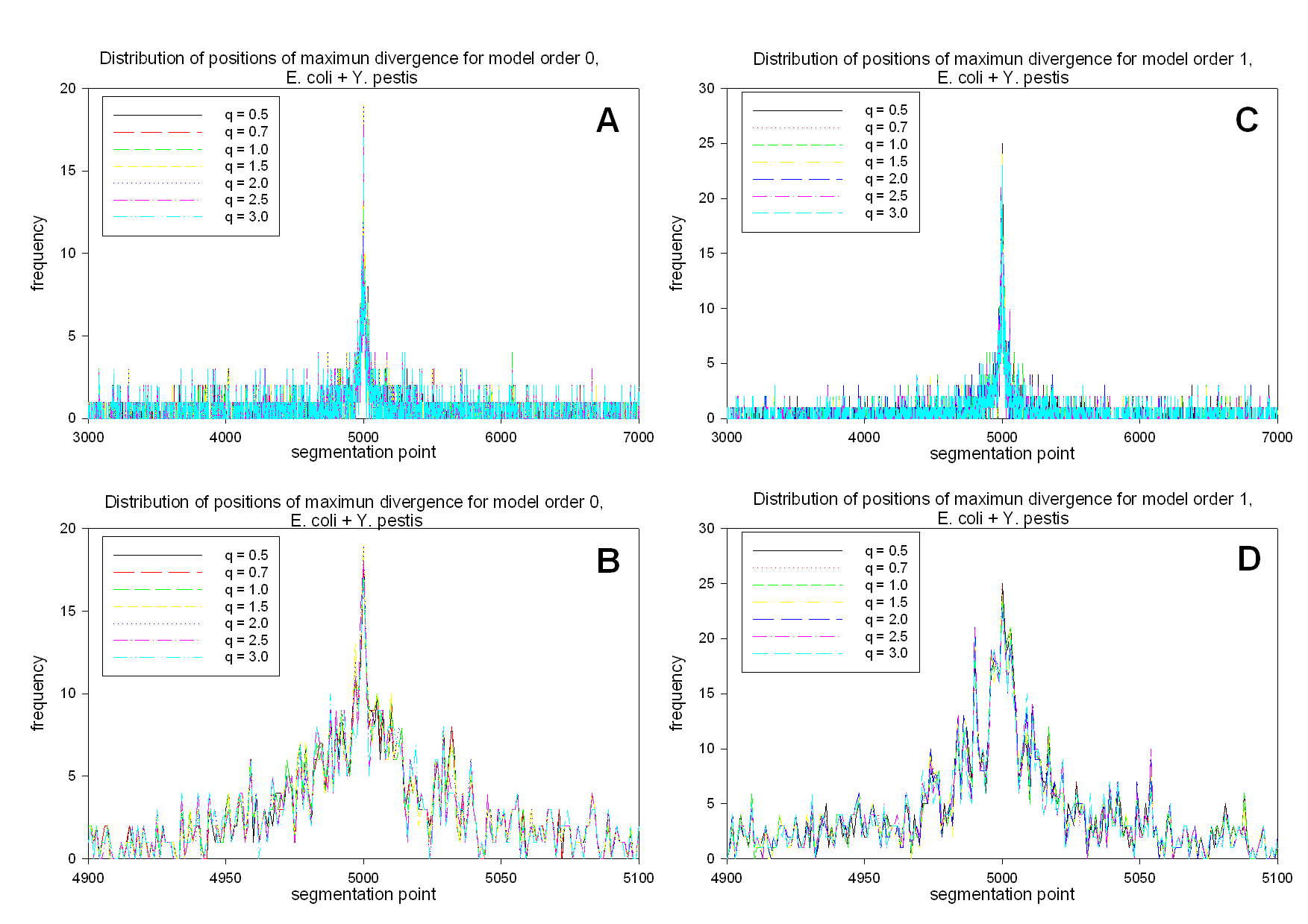

Supplement: Figure S28 — Frequency distribution of position with maximum value of non-extensive MJSD for the chimeric sequence constructs E. coli Y. pestis, for model order m = 0 (A, B) and 1 (C, D). For each model order, distributions are shown for different values of Tsallis statistics’ parameter q, in the range 0.5–3. The chimeric constructs of size 20 Kbp are comprised of two sequences, one component sequence of length 5 Kbp obtained from the genome of Y. pestis and the other of length 15 Kbp from the genome of E. coli. (TIF) [file pone.0093532.s028.tif]

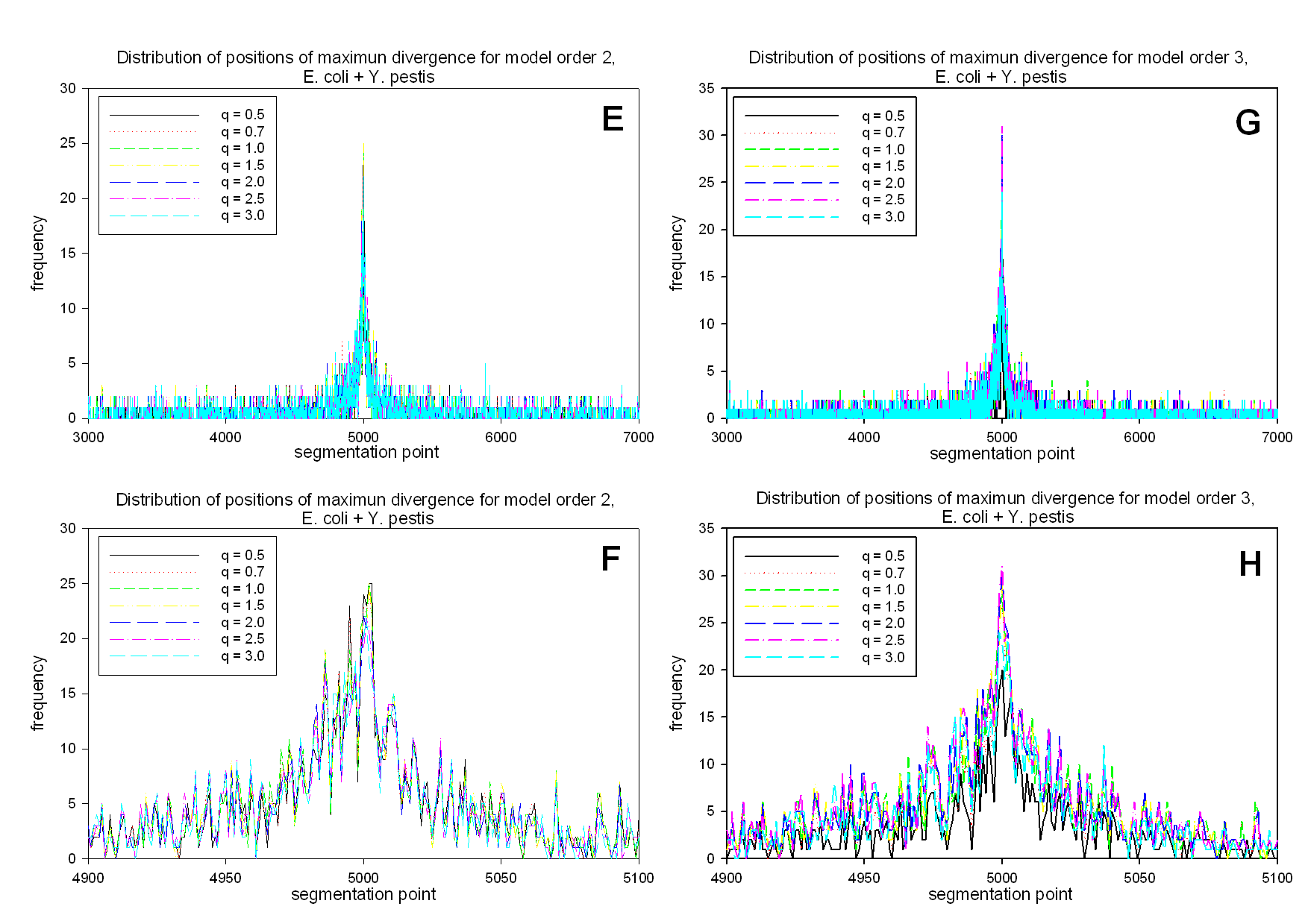

Supplement: Figure S29 — As in Figure S28, but for model order m = 2 (E, F) and 3 (G, H). (TIF) [file pone.0093532.s029.tif]

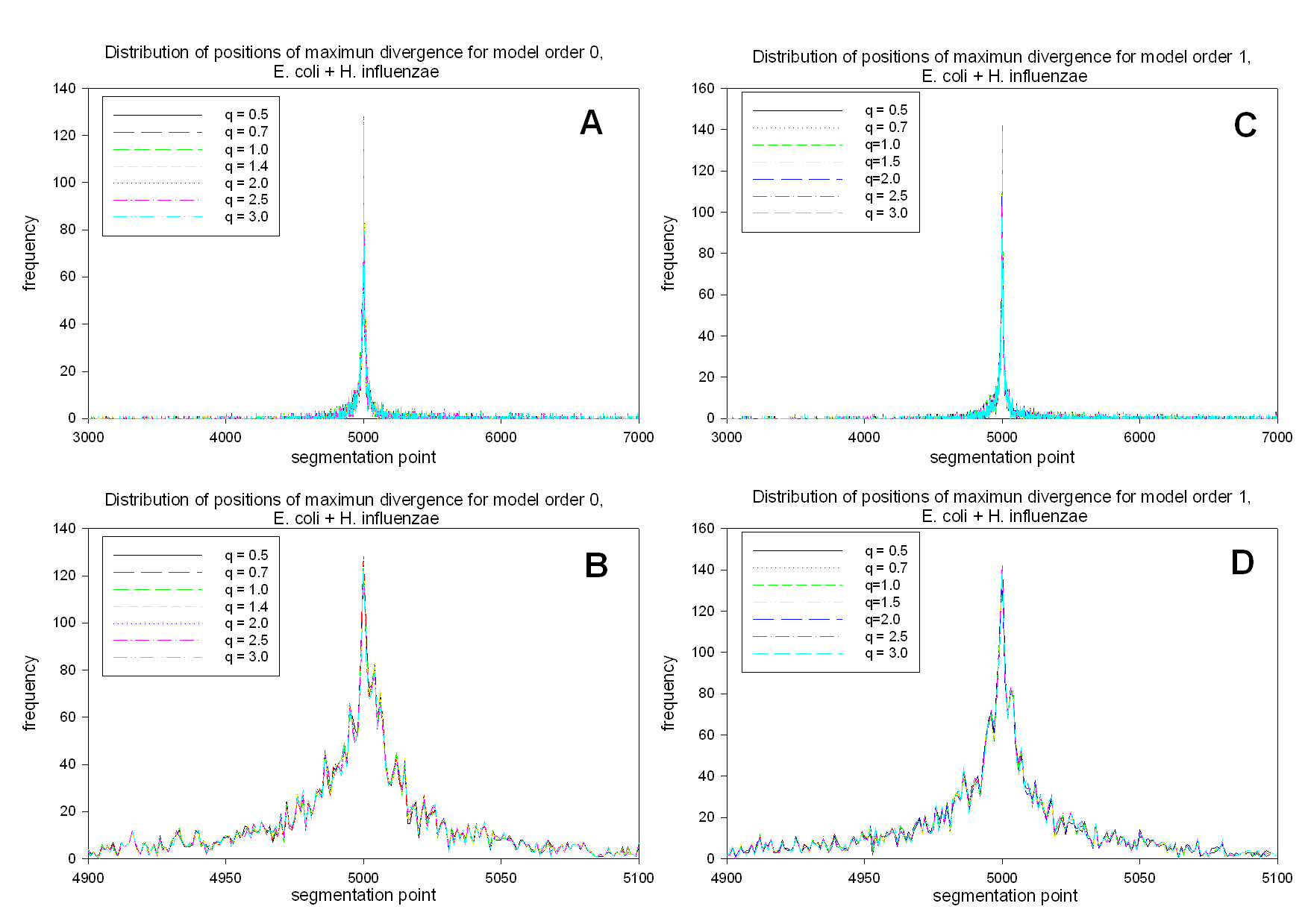

Supplement: Figure S30 — Frequency distribution of position with maximum value of non-extensive MJSD for the chimeric sequence constructs E. coli H. influenzae, for model order m = 0 (A, B) and 1 (C, D). For each model order, distributions are shown for different values of Tsallis statistics’ parameter q, in the range 0.5–3. The chimeric constructs of size 20 Kbp are comprised of two sequences, one component sequence of length 5 Kbp obtained from the genome of H. influenzae and the other of length 15 Kbp from the genome of E. coli. (TIF) [file pone.0093532.s030.tif]

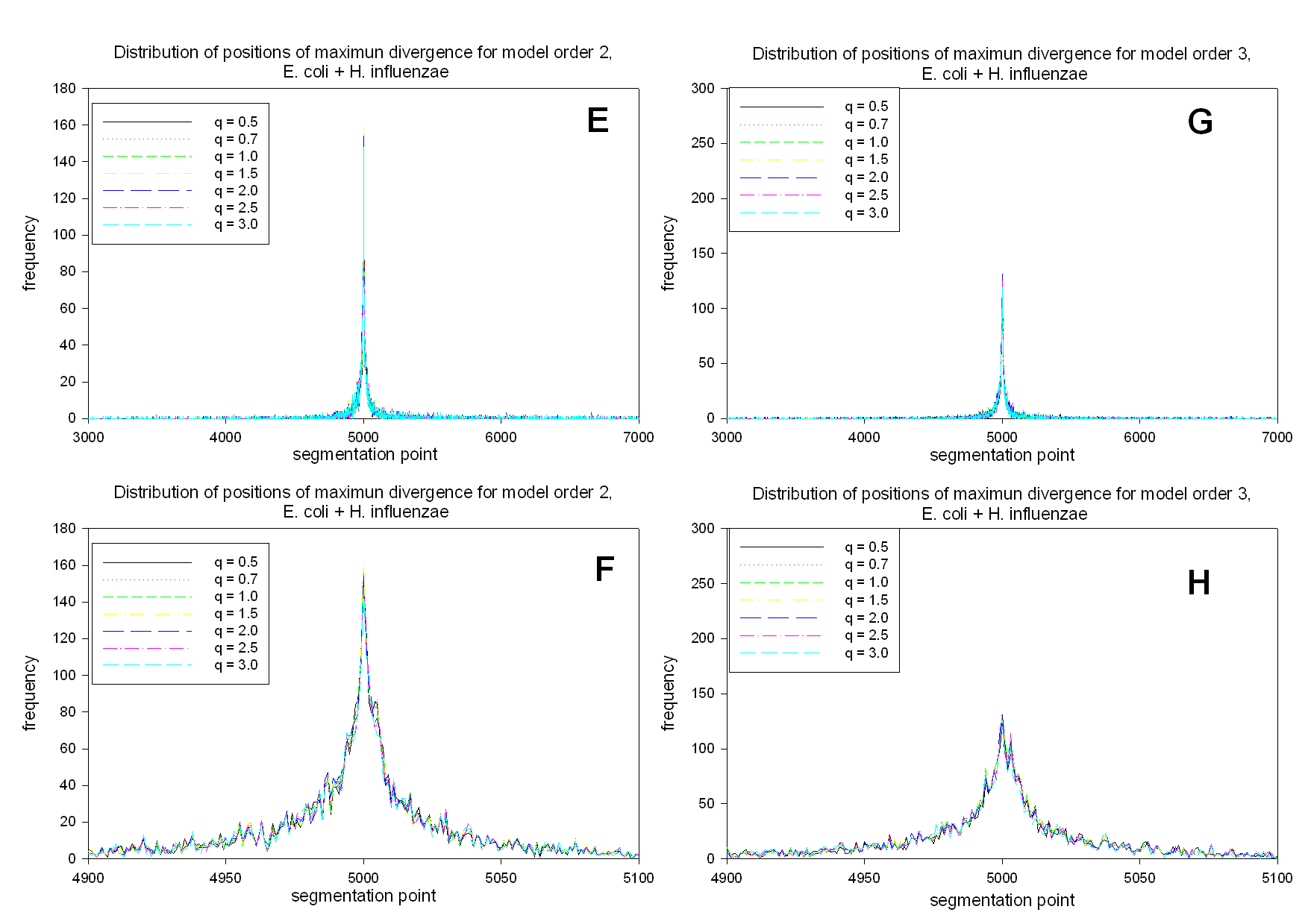

Supplement: Figure S31 — As in Figure S30, but for model order m = 2 (E, F) and 3 (G, H). (TIF) [file pone.0093532.s031.tif]
